# A Cation-Driven Approach toward Deep-Ultraviolet Nonlinear Optical Materials

**DOI:** 10.34133/research.0053

**Published:** 2023-03-09

**Authors:** Cong Hu, Meng Cheng, Wenqi Jin, Jian Han, Zhihua Yang, Shilie Pan

**Affiliations:** ^1^ Research Center for Crystal Materials, CAS Key Laboratory of Functional Materials and Devices for Special Environments, Xinjiang Technical Institute of Physics & Chemistry, CAS, 40–1 South Beijing Road, Urumqi 830011, China.; ^2^ Center of Materials Science and Optoelectronics Engineering, University of Chinese Academy of Sciences, Beijing 100049, China.

## Abstract

The design of new materials with special performances is still a great challenge, especially for the deep-ultraviolet nonlinear optical materials in which it is difficult to balance large bandgaps and strong second harmonic generation responses due to their inverse relationship. Cation variation not only influences the whole structure frameworks but also directly participates in the formation of electronic structures, both of which could lead to the uncontrollability of the properties of the designed materials. Here, a novel approach, aiming at purposeful and foreseeable material designs, is proposed to characterize the role of cations. By the verification of several series of borates, the influences of cation variation on property changes are explored systematically. Accordingly, a feasible strategy of designing deep-ultraviolet nonlinear optical materials by substituting barium for lead has been concluded, which could obviously blue-shift the ultraviolet cutoff edge and maintain the relatively strong second harmonic generation response (more than 2 times of KH_2_PO_4_), achieving the property optimization, and especially works efficiently in fluorooxoborates. The property optimization design strategy and the cation characterization method are not only helpful in exploring nonlinear optical materials but also enlightening in material design and selection.

## Introduction

Aiming at desirable performances, the material exploration assisted by quantum chemical calculation on computers has led to many new approaches, such as high-throughput screening and machine learning in data mining, or structure prediction by exploratory algorithms [1,2]. With the great demand in laser photolithography, micro-nano processing, optical communication, and medical treatment [3–10], a series of nonlinear optical (NLO) crystals have been discovered and commercialized successfully [11–17]. At present, deep-ultraviolet (DUV) NLO materials particularly attract many academic and commercial interests as the frequency conversion core to achieve coherent light with large photon flow and ultrahigh resolution and are expected to break the “200 nm wall”[18]. However, a large bandgap (*E*_g_ > 6.2 eV) is indispensable for DUV transparency, while *E*_g_ is inversely proportional to the second harmonic generation (SHG) response (*d*_ij_) [19]. Therefore, how to satisfy the balance between the two critical performances *(E*_g_ and *d*_ij_) is a key factor in the design of outstanding DUV NLO materials. KBe_2_BO_3_F_2_ (KBBF) exhibits excellent properties as a promising DUV NLO crystal [20,21], but the toxicity and layer-growing habit seriously impede its application. Finding KBBF replacements and balancing *E*_g_ and *d*_ij_ in the DUV region are still urgent.

To guarantee appropriate *E*_g_ and *d*_ij_ of designed NLO materials, several advantageous NLO-active (the sources of NLO effects) anion units are introduced, such as conjugated π configurations [BO_3_]^3−^, [B_3_O_6_]^3−^, distorted d^0^ or d^10^ transition-metal-centered (V^5+^, Cd^2+^) polyhedra, fluorooxoborate anionic units [BO*_n_*F_4-*n*_]^(*n*+1)−^ (*n* = 1, 2, 3), and so on [22–25]. Actually, as parts of the whole electron structures, A-site metal cations could also directly influence the optical properties besides the size effects in crystal structures, but a special study of the role of cation variation is rare. Here, a convenient cation characterization approach, “Cation differentiation by electron spatial distribution” (CRESD), is proposed to distinguish the role of cations in optical properties, and an accompanying design strategy is concluded from cation variation to achieve property optimization, for example, balancing large *E*_g_ and large *d*_ij_ in the DUV region. We note that the property optimization strategy and cation characterization approach can be applied in the design of high-performance materials not only for the NLO functions but also for other objects, such as perovskite solar cells.

## Results and Discussion

### Design strategy based on cations

**Fig. 1.**
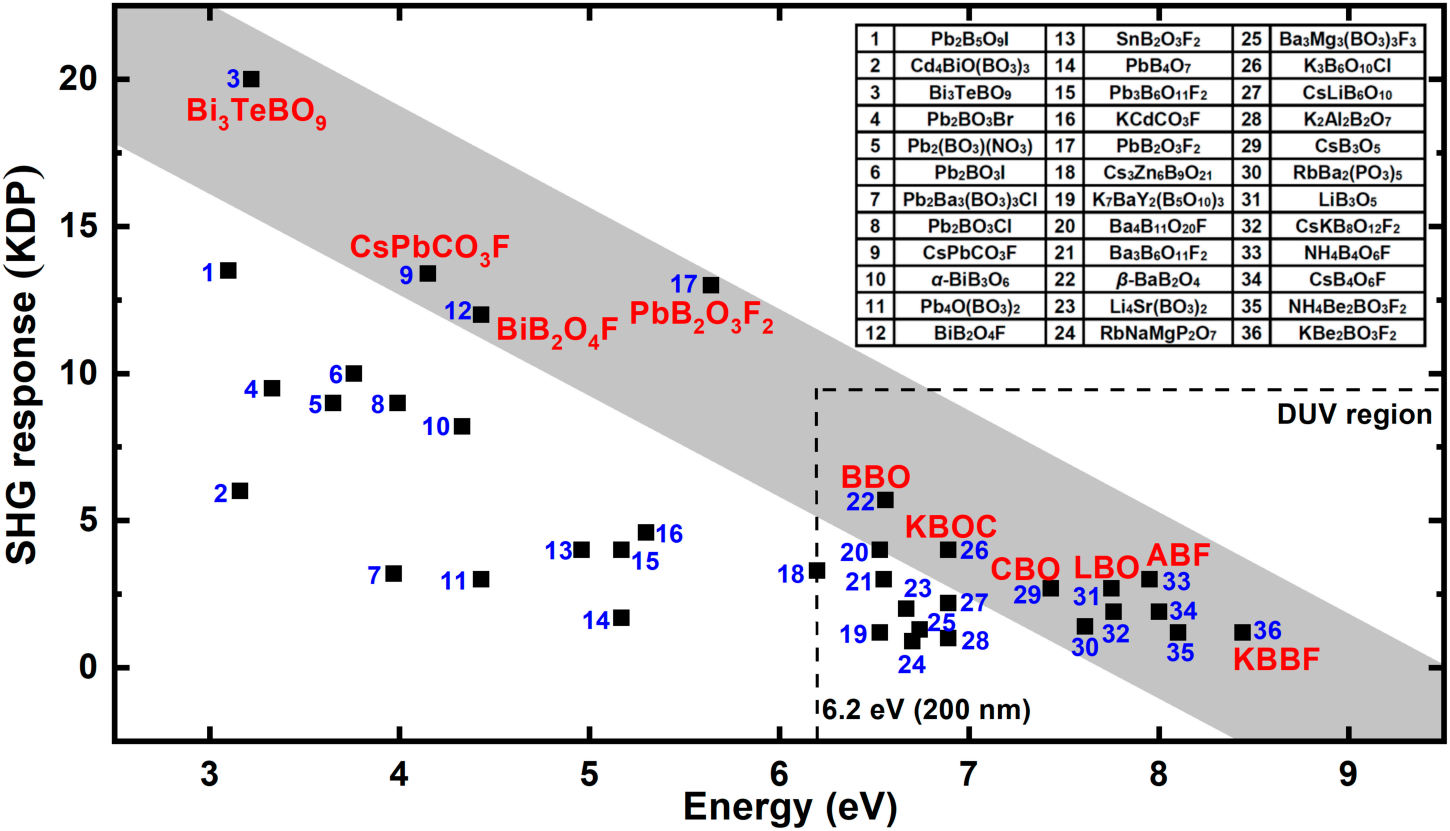
Comparison of typical NLO materials on bandgap and SHG response. Nonlinear optical (NLO); second harmonic generation (SHG). Several famous NLO crystals and promising materials with outstanding SHG responses are highlighted in the different bandgap regions: (I) 3 to 4 eV, Bi_3_TeBO_9_ (20.0 KDP); (II) 4 to 5 eV, CsPbCO_3_F (13.4 KDP) and BiB_2_O_4_F (12.0 KDP); (III) 5 to 6 eV, PbB_2_O_3_F_2_ (13.0 KDP); (IV) 6 to 7 eV, BBO (5.7 KDP) and K_3_B_6_O_10_Cl (4.0 KDP); (V) >7 eV, CBO (2.7 KDP), LBO (2.7 KDP), ABF (3.0 KDP), and KBBF (1.2 KDP).

Along with the increase of *E*_g_, the SHG response of typical NLO materials [26–50] demonstrates an obvious downward trend as shown in Fig. 1 (more details are shown in Table S1 in the Supplementary Materials). It is noteworthy that the compounds with outstanding SHG response (compared to *d*_36_ = 0.39 pm/V of KH_2_PO_4_ [KDP]) in different bandgap regions (or ultraviolet [UV] cutoff ranges) are highlighted in Fig. 1, especially, (I) 3 to 4 eV, Bi_3_TeBO_9_ (20.0 KDP) [51]; (II) 4 to 5 eV, CsPbCO_3_F (13.4 KDP) [52] and BiB_2_O_4_F (12.0 KDP) [53]; (III) 5 to 6 eV, PbB_2_O_3_F_2_ (13.0 KDP) [54]; (IV) 6 to 7 eV, *β*-BaB_2_O_4_ (BBO, 5.7 KDP) [55] and K_3_B_6_O_10_Cl (KBOC, 4.0 KDP) [56]; and (V) > 7 eV, CsB_3_O_5_ (CBO, 2.7 KDP) [57], LiB_3_O_5_ (LBO, 2.7 KDP) [58], NH_4_B_4_O_6_F (ABF, 3.0 KDP) [59], and KBBF (1.2 KDP) [21]. Among the above materials, four compounds exhibit a remarkably large SHG response, more than 10 times that of KDP, and half of the four ones contain lead atoms. However, commercialized applications of lead-containing NLO materials would face two shortcomings, narrow bandgaps, which is one of the main obstacles to the practical DUV applications, and the nonnegligible toxicity of lead cations. The unbalanced performances drive the combination properties of lead borates to be optimized.

**Fig. 2.**
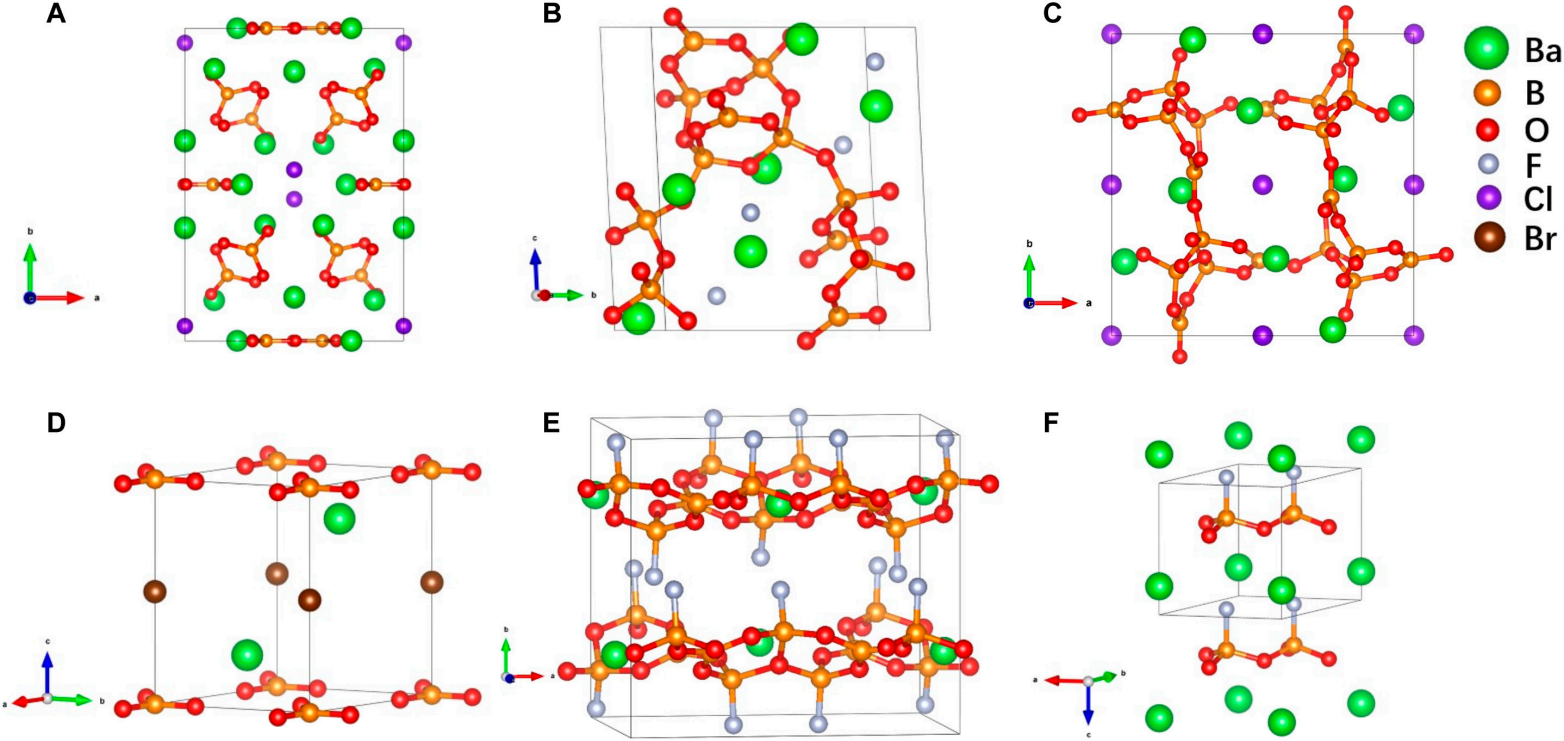
Crystal structures of barium borates after cation substitutions, Ba_5_(BO_3_)_3_Cl (A), Ba_3_B_6_O_11_F_2_ (B), Ba_2_B_5_O_9_Cl (C), Ba_2_BO_3_Br (D), BaB_5_O_7_F_3_ (E), and BaB_2_O_3_F_2_ (*P*31*m*) (F).

To verify the feasibility of property optimization by the design strategy of cations, six lead borates with hierarchical SHG responses, from 3.5 to 13.4 KDP, are selected: Pb_2_Ba_3_(BO_3_)_3_Cl [31], Pb_3_B_6_O_11_F_2_ [35], Pb_2_B_5_O_9_Cl [60], Pb_2_BO_3_Br [28], PbB_5_O_7_F_3_ [61], and PbB_2_O_3_F_2_ [54], which crystallize in the noncentrosymmetric space groups, *C*222_1_ (No. 20), *P*2_1_ (No. 4), *Pnn*2 (No. 34), *P*321 (No. 150), *Cmc*2_1_ (No. 36), and *P*31*m* (No. 157), respectively. The isostructural barium borates of the first 3 have been reported previously, namely, Ba_5_(BO_3_)_3_Cl [62], Ba_3_B_6_O_11_F_2_ [40], and Ba_2_B_5_O_9_Cl [63]. It notes that between Pb_2_Ba_3_(BO_3_)_3_Cl and Ba_5_(BO_3_)_3_Cl, the substitution from Pb^2+^ to Ba^2+^ only exists in the 8*c* cation Wyckoff positions, while Ba^2+^ in two 4*b* and one 4*a* positions are retained. In Pb_2_Ba_3_(BO_3_)_3_Cl/Ba_5_(BO_3_)_3_Cl, the anionic framework is formed by noncondensed BO_3_ units, while both Pb_2_B_5_O_9_Cl/Ba_2_B_5_O_9_Cl and Pb_3_B_6_O_11_F_2_/Ba_3_B_6_O_11_F_2_ possess a 3-dimensional B-O network consisting of BO_3_ and BO_4_ units (Fig. 2A to C). Theoretical structural models Ba_2_BO_3_Br, BaB_5_O_7_F_3_, and BaB_2_O_3_F_2_ (BaB_2_O_3_F_2_-*P*31*m*, hereinafter referred to as BaB_2_O_3_F_2_) are derived from Pb_2_BO_3_Br, PbB_5_O_7_F_3_, and PbB_2_O_3_F_2_ by cation substitutions, in which the anion frameworks are formed by a noncondensed BO_3_ group, a [B_5_O_7_F_3_]_∞_ layer, and a [B_6_O_9_F_6_]_∞_ layer, respectively. It should be noticed that the connection of the halide atoms is also different, where F in the fluorooxoborate unit BO_3_F connects both with the metal Ba and B atoms, while Br connects only to the metal Ba (Fig. 2D to F). The structural information of predicted Ba_2_BO_3_Br, BaB_5_O_7_F_3,_ and BaB_2_O_3_F_2_ are shown in Tables S2–S4, and their crystallographic details (atomic coordinates, bond distances, angles, etc.) have been listed in Tables S5–S12.

**Fig. 3.**
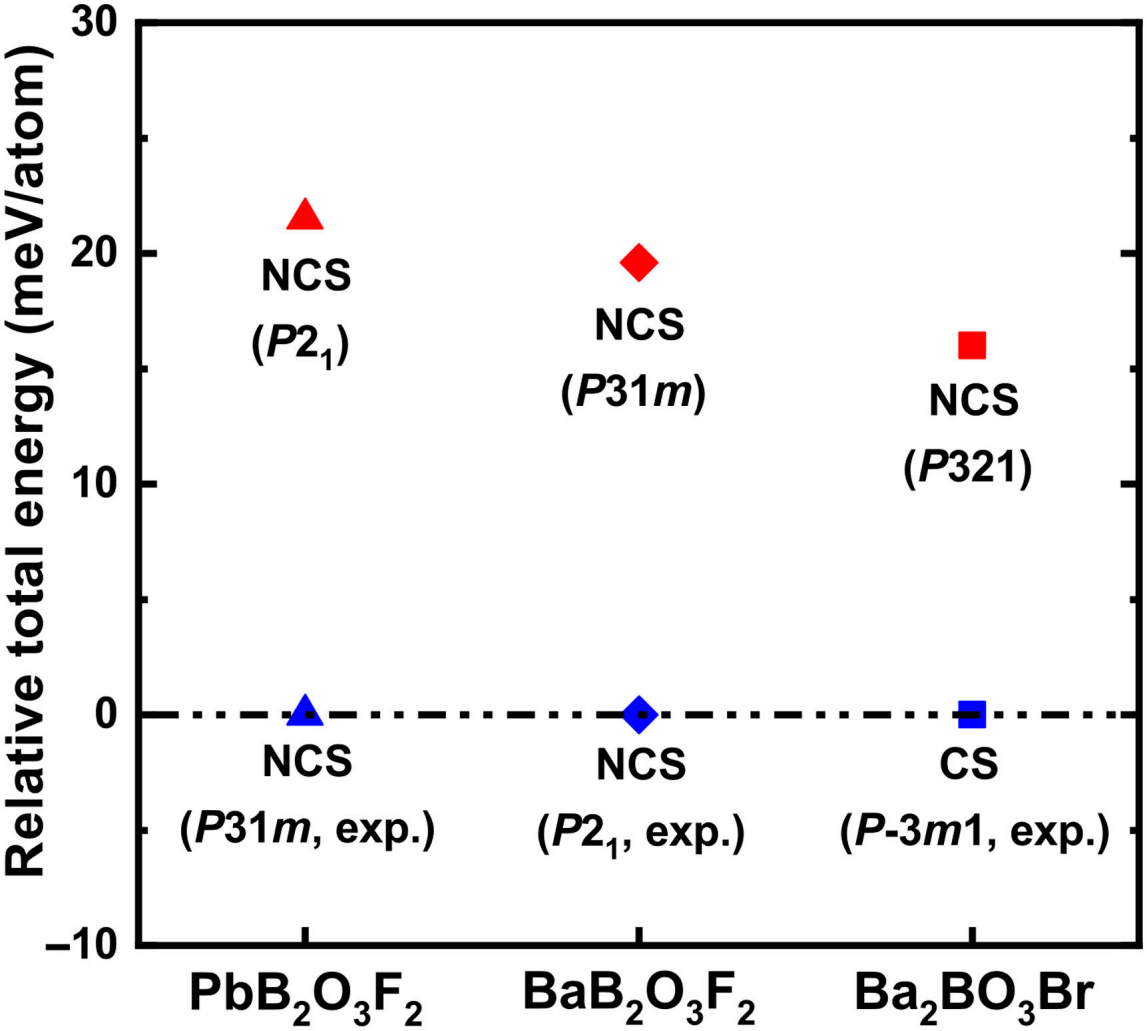
Relative total energy between theoretical (red) and experimental (blue) structures in PbB_2_O_3_F_2_, BaB_2_O_3_F_2_, and Ba_2_BO_3_Br.

In addition, recently, a noncentrosymmetric BaB_2_O_3_F_2_ with lower symmetry, *P*2_1_, was successfully synthesized by our group [64]. The relative total energy (Helmholtz free energy) calculated by DFT (density functional theory) between predicted BaB_2_O_3_F_2_ (*P*31*m*) and experimental BaB_2_O_3_F_2_ (*P*2_1_) is less than 20 meV/atom (Fig. 3), and the formation enthalpy of BaB_2_O_3_F_2_ (*P*31*m*) is 0.024 eV/atom, and there is no imaginary phonon mode in the phonon spectrum of BaB_2_O_3_F_2_ (*P*31*m*) (Fig. S1), indicating the thermodynamical metastability and kinetical stability. Moreover, for comparison, the structure of PbB_2_O_3_F_2_ (*P*2_1_) is built by a cation substitution for experimental BaB_2_O_3_F_2_ (*P*2_1_), and the relative total energy between PbB_2_O_3_F_2_ (*P*2_1_) and experimental PbB_2_O_3_F_2_ (*P*31*m*) is about 20 meV/atom, and the formation enthalpy of PbB_2_O_3_F_2_ (*P*2_1_) is −0.023 eV/atom, which shows the thermodynamical possibility of being synthesized. Here, the formation enthalpies of BaB_2_O_3_F_2_ (*P*31*m*) and PbB_2_O_3_F_2_ (*P*2_1_) are calculated from the general experimental source, B_2_O_3,_ and BaF_2_/PbF_2_.

### CRESD method

To clarify the role of cations in property changes, the CRESD method is proposed to characterize the volumetric effects of various metal cations by the occupied volume of electrons, namely, electron spatial distribution (ESD). Taking the above isostructural substitutions of lead/barium borates as examples, the magnitudes of ESD between lead and barium atoms are compared on the basis of atom radius R(M), cation radius R(M^2+^), and cation volume V(M^2+^), where V(M^2+^) = (2 × R(M^2+^))^3^ (Table 1). Here, deviation γ is defined as (*P*_1_ − *P*_2_)/((*P*_1_+*P*_2_)/2) to evaluate the differentiation of *P*_1_ and *P*_2_, where parameter *P* could be the magnitudes of ESD based on atom/cation radius and cation volume, or any other parameters, such as crystal volume, in the following contents. The deviations γ of Pb^2+^/Ba^2+^ ESD based on R(M), R(M^2+^), and V(M^2+^) are 21.4%, 13.3%, and 39.5%, respectively. Considering the isostructural substitutions, the deviation γ of ESD between Pb^2+^ and Ba^2+^ should change slightly. ESD [R(M^2+^)] is better to characterize the cations but hard to sum arithmetically for composite cations. ESD [V(M^2+^)] can also be summed arithmetically, but its deviation γ is too large. More importantly, all of the above ESD values are incapable of ion groups, such as [NH_4_]^+^, [CH_3_NH_3_]^+^, and so on. Consequently, ESD based on Bader volumes could overcome the above restrictions, where the electron density in the whole space is partitioned by DFT calculation [65,66], and hereinafter the ESD refers to the results on Bader volumes. Pb^2+^ and Ba^2+^ have similar values of ESD, and their deviations γ are 6.7%, 8.7%, and 9.0% in M_2_Ba_3_(BO_3_)_3_Cl, M_3_B_6_O_11_F_2_, and M_2_B_5_O_9_Cl (M = Pb^2+^, Ba^2+^), all of which are lower than that of ESD [R(M^2+^)] (Table 2), indicating that the ESD changes slightly in isostructural substitution.

**Table 1.** Comparison between lead and barium elements of ESD based on atom radius R(M), valence state radius R(M^2+^), and ionic volume V(M^2+^).

	ESD[R(M)] (Å)	ESD[R(M^2+^)] (Å)	ESD[V(M^2+^)] ^b^ (Å^3^)
Pb	1.75	1.19	13.5
Ba	2.17	1.36	20.1
Deviation γ ^a^	21.4%	13.3%	39.5%

^a^Deviation γ, for example, is defined as $\gamma =\frac{P\left(\mathrm{Ba}\right)-P\left(\mathrm{Pb}\right)}{\left(P\left(\mathrm{Ba}\right)+P\left(\mathrm{Pb}\right)\right)/2}$, where *P* is the magnitude of ESD based on radius R or volume V. Electron spatial distribution (ESD).

^b^ESD based on ionic volume is calculated by ESD[V(M^2+^)] = (2 × R(M^2+^))^3^

**Table 2.** Comparison between lead and barium compounds of cation ESD based on Bader volumes.

Compound A	ESD (Å^3^)	Compound B	ESD (Å^3^)	Deviation γ
Pb_2_Ba_3_(BO_3_)_3_Cl	23.1 (Pb1)	Ba_5_(BO_3_)_3_Cl	24.7 (Ba1)	6.7%
				
Pb_3_B_6_O_11_F_2_	22.6 (Pb1)	Ba_3_B_6_O_11_F_2_	24.2 (Ba1)	6.8%
	22.0 (Pb2)		24.0 (Ba2)	8.7%
	21.3 (Pb3)		23.7 (Ba3)	10.7%
			Average	8.7%
				
Pb_2_B_5_O_9_Cl	22.7 (Pb1)	Ba_2_B_5_O_9_Cl	24.2 (Ba1)	6.4%
	22.0 (Pb2)		24.7 (Ba2)	11.6%
			Average	9.0%
				
Pb_2_BO_3_Br	28.3 (Pb1)	Ba_2_BO_3_Br	32.5 (Ba1)	13.9%
				
PbB_5_O_7_F_3_	21.5 (Pb1)	BaB_5_O_7_F_3_	24.2 (Ba1)	11.8%
				
PbB_2_O_3_F_2_	22.0 (Pb1)	BaB_2_O_3_F_2_	23.3 (Ba1)	5.7%

ESD can be not only applied in the comparison of experimental structures but also introduced to analyze the change regulations on structures and performances of theoretical structures, such as Ba_2_BO_3_Br and BaB_2_O_3_F_2_ mentioned above. As the lattice parameters are relaxed in geometric structure optimization, the values of ESD have been calculated from both optimized and experimental (no-optimization) structures. The ESD and crystal volume in Pb_2_BO_3_Br are 27.7/28.3 and 143.6/149.0 Å^3^ without/with optimization, and their deviation γ is 2.2% and 3.7% (Table S2), indicating the feasibility of structure optimization. Interestingly, the ESD of Pb/Ba in Pb_2_BO_3_Br/Ba_2_BO_3_Br is 28.3/32.5 Å^3^, distinctly larger than those of the other 4 couples of lead/barium borates, ~22.0/24.0 Å^3^, and the deviation γ of cation ESD is 13.9%, the largest one among the 6 couples of lead/barium borates. The reason mainly comes from incompact structures. Pb_2_BO_3_Br/Ba_2_BO_3_Br belongs to rich metal borates with metal:boron = 2:1, resulting in the delocalization of electrons around metals, where the evidence is the larger deviation γ of crystal volumes between Pb_2_BO_3_Br and Ba_2_BO_3_Br, 18.6%. On the contrary, PbB_2_O_3_F_2_/BaB_2_O_3_F_2_ contains fewer metal atoms, metal:boron = 1:2, and the cation ESD is also relatively small, 22.0/22.3 Å^3^. The deviation γ of ESD is the smallest, 5.7%, and the deviation γ of crystal volumes is only 3.0% (Table S3).

### Property optimization for balancing bandgaps and SHG responses

To assess the influence of cation variation on property change, the optical properties of the selected lead/barium borates are evaluated and analyzed in detail by DFT calculation. Based on generalized gradient approximation (GGA)-Perdew-Burke-Ernzerhof (PBE) functionals, the calculated bandgaps of these lead borates, Pb_2_Ba_3_(BO_3_)_3_Cl, Pb_3_B_6_O_11_F_2,_ and Pb_2_B_5_O_9_Cl, are 3.54, 3.81, and 3.36 eV, which are lower than their experimental or HSE06 values, 3.97 eV (312 nm), 5.17 eV (240 nm), and 4.41 eV (281 nm, HSE06), respectively. Here, the underestimation of bandgaps under GGA-PBE functional is derived from the inherent discontinuous exchange-correlation energy [67], and a scissors operator is adopted as the difference between the GGA bandgap and the experimental bandgap to eliminate this underestimation in the calculation of optical properties. In addition, a more expensive calculation, hybrid functional HSE06 [68], is used to achieve the value of bandgap more accurately for the lack of experimental bandgaps.

**Fig. 4.**
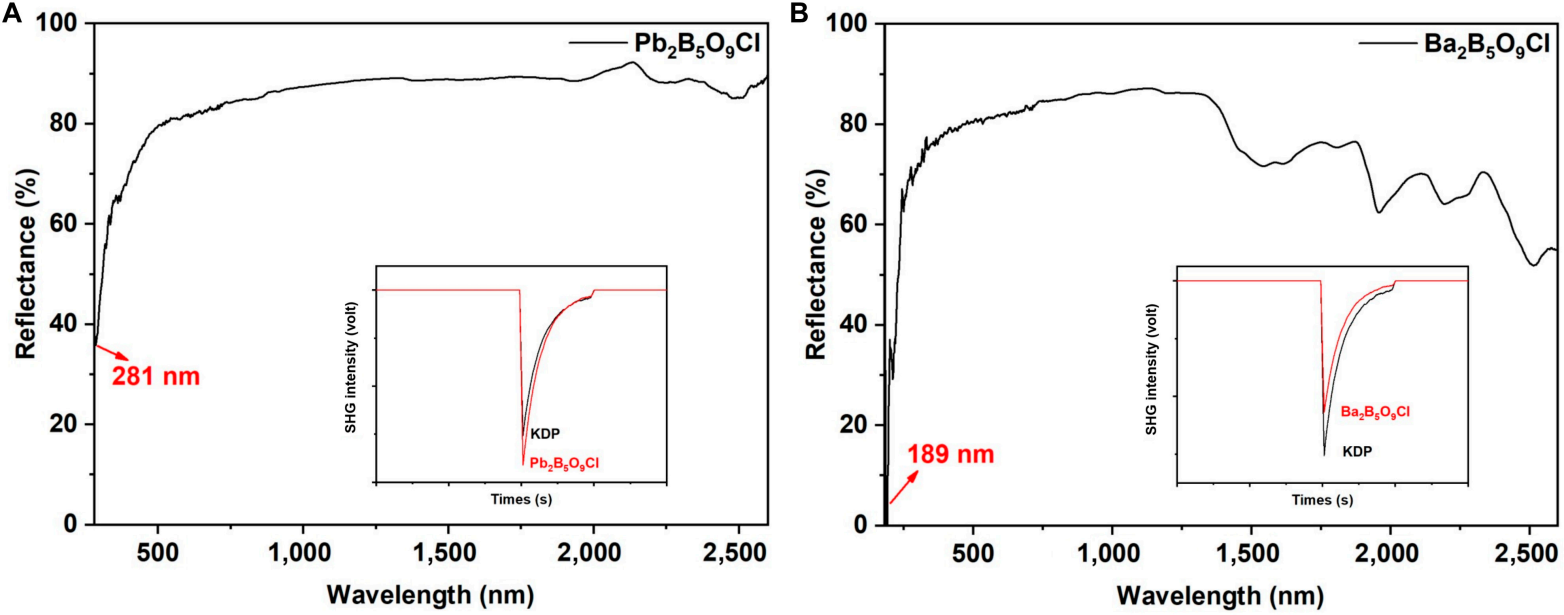
Ultraviolet−visible−near infrared diffuse reflectance spectrum of Pb_2_B_5_O_9_Cl (A) and Ba_2_B_5_O_9_Cl (B). The insets are SHG intensity oscilloscope traces.

To verify the theoretic simulations, a couple of borates, M_2_B_5_O_9_Cl (M = Pb, Ba), were synthesized in experiments, and the UV cutoff edge and SHG intensity were also measured (powder X-ray diffraction [XRD] patterns are shown in Fig. S2). As shown in Fig. 4, the UV absorbing cutoff of Pb_2_B_5_O_9_Cl is 281 nm, which is consistent with our HSE06 calculation value of 281 nm. Ba_2_B_5_O_9_Cl shows a UV cutoff edge of 189 nm, which is similar to 185 nm in the previous work [63].

By cation substitution in the three lead borate prototypes with moderate SHG responses, the bandgaps of barium borates have increased to 5.26 eV (236 nm, HSE06), 6.53 eV (190 nm, exp.), and 6.56 eV (189 nm, exp., this work) for Ba_5_(BO_3_)_3_Cl, Ba_3_B_6_O_11_F_2,_ and Ba_2_B_5_O_9_Cl, which exhibit 76, 50, and 92 nm blue-shift from the isostructural lead borates. Correspondingly, the SHG responses have changed to 0.5, 3.0, and 0.7 KDP for Ba_5_(BO_3_)_3_Cl, Ba_3_B_6_O_11_F_2_, and Ba_2_B_5_O_9_Cl from 3.2, 4.0, and 1.2 KDP of Pb_2_Ba_3_(BO_3_)_3_Cl, Pb_3_B_6_O_11_F_2_, and Pb_2_B_5_O_9_Cl. The experimental SHG intensities of M_2_B_5_O_9_Cl (M = Pb, Ba) are shown in the insets of Fig. 4. It is noted that both Ba_3_B_6_O_11_F_2_ and Ba_2_B_5_O_9_Cl have obtained DUV cutoff edges (<200 nm), but only borate fluoride Ba_3_B_6_O_11_F_2_ maintained relatively strong SHG responses (> 2 KDP). For the last three prototypes, borate halide Pb_2_BO_3_Br and fluorooxoborates PbB_5_O_7_F_3_ and PbB_2_O_3_F_2_ exhibit remarkably strong SHG responses, 9.5, 6.0, and 13.0 KDP, but their bandgaps are different, 3.33 eV (372 nm), 5.51 (225 nm), and 5.64 eV (220 nm), respectively. After cation substitutions, the combination property of the former changes slightly, 3.87 eV (320 nm) and 7.8 KDP for Ba_2_BO_3_Br. Contrarily, BaB_5_O_7_F_3_ and BaB_2_O_3_F_2_ show a great change in their prototype, 7.92 eV (157 nm) with 2.5 KDP, and 8.24 eV (150 nm) with 4.8 KDP. Furthermore, to analyze the source of the variation in bandgaps among the above metal borates, the total and partial density of states are shown in Figs. S3 to S8. Compared to the partial density of states among these metal borates, it is found that the conduction band minima are mainly composed of Pb-p or Ba-d and a little of B-p orbitals, while the valence band maxima mainly contain O-p, a little of halogen p orbitals, along with slight Pb/Sn-s orbitals for the stereochemically active lone pairs. According to the revised lone pair model [69], the interaction between Pb and O drives from the mixture of empty Pb p and a filled antibonding state between Pb-s and O-p states in PbO, and the lone pair stereochemical activity of Pb cations greatly influences the reduction of bandgaps in series of metal borates [70]. Therefore, the cation substitution from Pb to Ba could avoid the stereochemical activity of the lone pairs to achieve a relatively large bandgap, a similar effect that introducing the F atom could eliminate the dangling bonds of BO_3_ groups in a series of fluorooxoborates [25,47,50,59]. As far as the purpose of property optimization, doubtless, the cation substitution in the fluorooxoborates BaB_5_O_7_F_3_ and BaB_2_O_3_F_2_ is more successful.

**Fig. 5.**
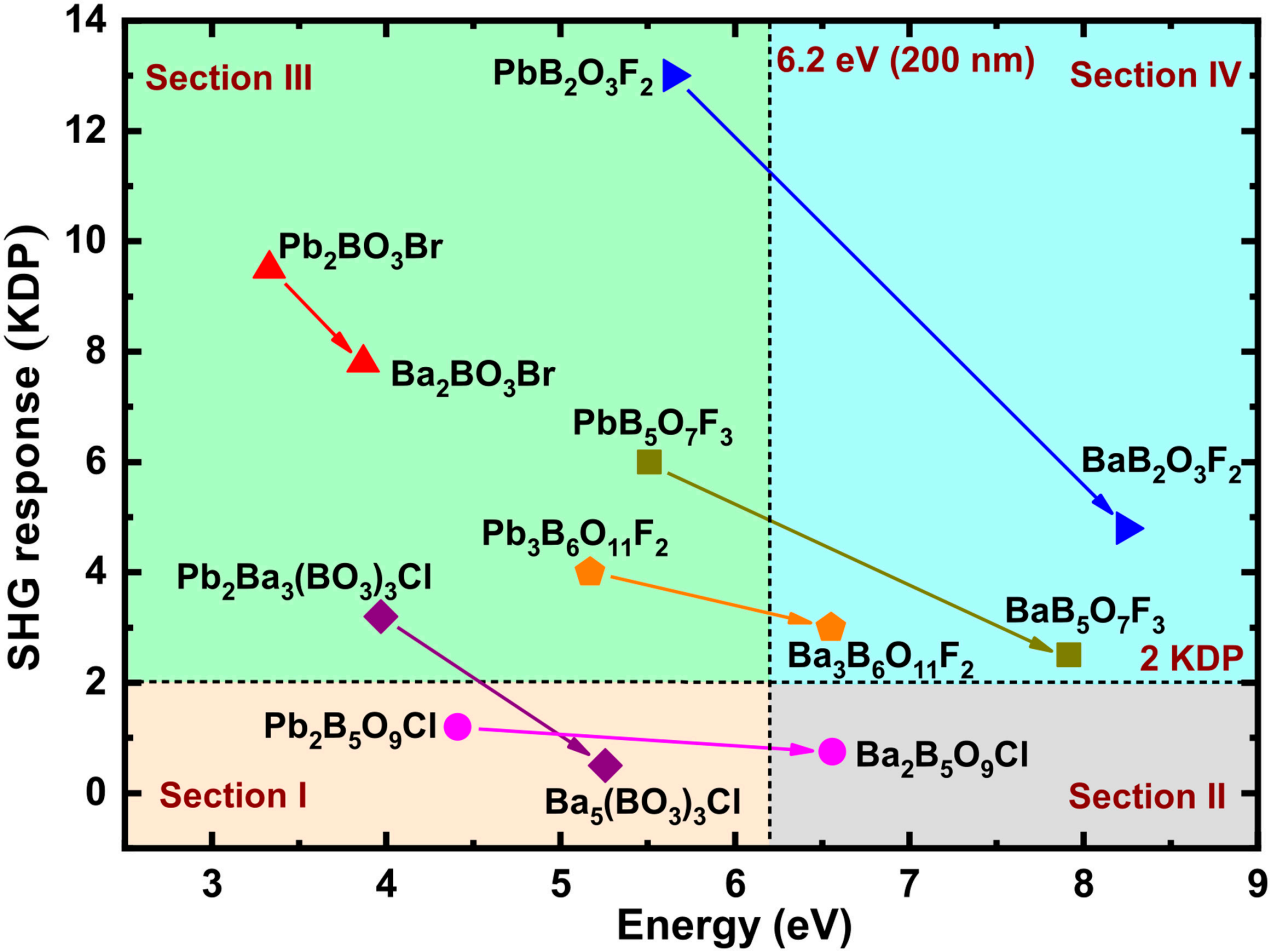
Property changes from lead borates to barium borates. Changes in optical properties (SHG response and bandgap), and the classifications of four value sections based on the DUV (200 nm) and relatively strong SHG response (2 KDP) bounds in the cation substitution from lead to barium. Deep-ultraviolet (DUV); second harmonic generation (SHG).

Figure 5 shows the changes for bandgap (*E*_g_) and SHG response in the cation substitution from lead to barium. The whole region is divided into 4 sections: I, *E*_g_ < 6.2 eV, SHG < 2 KDP; II, *E*_g_ ≥ 6.2 eV, SHG < 2 KDP; III, *E*_g_ < 6.2 eV, SHG ≥ 2 KDP; IV, *E*_g_ ≥ 6.2 eV, SHG ≥ 2 KDP, where 6.2 eV and 2 KDP are DUV and relatively strong SHG response bounds, respectively. Here, before the cation substitution, 5 lead borates in 6 are all located at Section III, indicating strong SHG responses but not DUV cutoff edges. After the cation substitution, these 5 barium borates scatter into 3 sections, 1 (Ba_2_BO_3_Br) still in Section III, 1 (Ba_5_(BO_3_)_3_Cl) in Section I, and 3 in Section IV (Ba_3_B_6_O_11_F_2_, BaB_5_O_7_F_3_, and BaB_2_O_3_F_2_). Although Ba_2_B_5_O_9_Cl shows a DUV bandgap, its SHG response is relatively small for the reason that the SHG intensity of its prototype is not strong. It is found that these lead borates could keep strong SHG responses (≥2 KDP) after cation substitution except Pb_2_Ba_3_(BO_3_)_3_Cl and Pb_2_B_5_O_9_Cl, whose SHG response is the smallest among the 6 lead borates. What is more important, the bandgaps of all the lead borates have visibly widened, and especially four of them have exceeded the DUV limit (6.2 eV, 200 nm) after cation substitution. Therefore, the cation substitution from lead to barium could optimize the combination properties.

Considering the importance of SHG response for NLO materials, exploring the role of cation variation on SHG response is vital to deepen the understanding of the structure–property relationship. The real-space atom-cutting technique is an efficient postprocessing tool to evaluate the contribution of microscopic structural groups to optical properties, such as the SHG coefficient. Table 3 shows the results of the calculated SHG coefficients before (original) and after (e.g., cut Pb) cutting the metal cations, and the “SHG reducing” is expressed as “SHG (reducing) = 1 − SHG (cut)/SHG (original)”, which is related to the contribution of metal cations to SHG coefficients. Various nonzero components of SHG coefficients are related to the different symmetries of the point groups (relatively small values, < 0.1 pm/V, are not listed here), and the final SHG response is characterized by the largest SHG coefficient in the effective SHG formula. The results indicate that in Ba_3_B_6_O_11_F_2_ and Ba_2_B_5_O_9_Cl, lead and barium cations contribute equally to the SHG coefficient. In M(BO_3_)_3_Cl (M = Pb_2_Ba_3_, Ba_5_), the Wyckoff site cations cause various contributions to the SHG coefficient *d*_14_. Namely, in Pb_2_Ba_3_(BO_3_)_3_Cl, two 8c Pb contribute the most of *d*_14_, 29% in 33%, and two 4b Ba and one 4a Ba contribute the relatively small parts, 7% and 0.3% in 33%, respectively. In Ba_5_(BO_3_)_3_Cl, two 8c Ba contribute almost all of the *d*_14_, but 4a Ba makes a remarkably negative contribution, −30%, which leads to an obvious decrease of the SHG response from Pb_2_Ba_3_(BO_3_)_3_Cl to Ba_5_(BO_3_)_3_Cl. For MB_5_O_7_F_3_ (M = Pb, Ba), the metal cations contribute about 10% to 15%, and the relatively small contribution mainly derives from the ratio of metal cations to B-O/B-O-F anion groups is only 1:5.

**Table 3.** Real-space atom-cutting results of SHG coefficients.

Compound		SHG Coefficient (pm/V)	SHG Reducing^a^	Compound		SHG Coefficient (pm/V)	SHG Reducing
Pb_2_Ba_3_(BO_3_)_3_Cl	Original	2.102 (*d*_14_)		Ba_5_(BO_3_)_3_Cl	Original	−0.481 (*d*_14_)	
	Cut PbBa	1.410 (*d*_14_)	33%		Cut Ba	−0.426 (*d*_14_)	11%
	Cut Pb (8c)	1.501 (*d*_14_)	29%		Cut Ba (8c)	−0.273 (*d*_14_)	43%
	Cut Ba (4b)	1.965 (*d*_14_)	7%		Cut Ba (4b)	−0.485 (*d*_14_)	−0.8%
	Cut Ba (4a)	2.095 (*d*_14_)	0.3%		Cut Ba (4a)	−0.627 (*d*_14_)	−30%
Pb_3_B_6_O_11_F_2_	Original	0.688 (*d*_14_) 2.057 (*d*_16_) −0.903 (*d*_22_)		Ba_3_B_6_O_11_F_2_	Original	−0.469 (*d*_14_) −1.104 (*d*_16_) 1.109 (*d*_22_)	
	Cut Pb	0.588 (*d*_14_) 1.150 (*d*_16_) −0.835 (*d*_22_)	14% 44% 8%		Cut Ba	−0.416 (*d*_14_) −0.680 (*d*_16_) 0.995 (*d*_22_)	11% 38% 10%
Pb_2_B_5_O_9_Cl	Original	−2.525 (*d*_15_) −0.811 (*d*_24_) 1.094 (*d*_33_)		Ba_2_B_5_O_9_Cl	Original	−0.742 (*d*_15_) −0.473 (*d*_24_) 0.766 (*d*_33_)	
	Cut Pb	−1.067 (*d*_15_) −0.688 (*d*_24_) 0.783 (*d*_33_)	58% 15% 28%		Cut Ba	−0.356 (*d*_15_) −0.409 (*d*_24_) 0.515 (*d*_33_)	52% 13% 33%
Pb_2_BO_3_Br	Original	6.182 (*d*_11_)		Ba_2_BO_3_Br	Original	3.039 (*d*_11_)	
	Cut Pb	1.708 (*d*_11_)	72%		Cut Ba	0.759 (*d*_11_)	75%
PbB_5_O_7_F_3_	Original	2.640 (*d*_15_) −1.341 (*d*_24_) −1.534 (*d*_33_)		BaB_5_O_7_F_3_	Original	1.010 (*d*_15_) −0.430 (*d*_24_) −0.747 (*d*_33_)	
	Cut Pb	2.273 (*d*_15_) −1.146 (*d*_24_) −1.288 (*d*_33_)	14% 15% 16%		Cut Ba	0.918 (*d*_15_) −0.375 (*d*_24_) −0.674 (*d*_33_)	9% 13% 10%
PbB_2_O_3_F_2_	Original	−2.800 (*d*_22_) −4.952 (*d*_33_)		BaB_2_O_3_F_2_	Original	−0.809 (*d*_22_) −1.881 (*d*_33_)	
	Cut Pb	−0.780 (*d*_22_) −2.776 (*d*_33_)	72% 44%		Cut Ba	−0.530 (*d*_22_) −0.732 (*d*_33_)	34% 61%
SnB_2_O_3_F_2_	Original	0.938 (*d*_22_) −0.849 (*d*_33_)					
	Cut Sn	0.254 (*d*_22_) −1.956 (*d*_33_)	73% −130%				

^a^SHG reducing is calculated by formula: SHG(reducing) = 1 − SHG(cut)/SHG(original)

Furthermore, in M_2_BO_3_Br_2_ and MB_2_O_3_F_2_ (M = Pb, Ba), the cations exert a remarkable contribution to the SHG responses. In addition, in the SHG density map (Fig. S9), there are plenty of unoccupied states contributing to the VE and virtual-hole (VH) process and occupied states contributing to the VH process around Pb and Ba. In MB_2_O_3_F_2_ (M = Pb, Sn, Ba), Sn shows a vitally negative contribution for *d*_33_ (−130%), which is the reason that the SHG response of SnB_2_O_3_F_2_ is much lower than that of isostructural PbB_2_O_3_F_2_, in accordance with the results from another group [54], while Sn and Pb play similar contributions to *d*_22_, 73% to 72%.

**Fig. 6.**
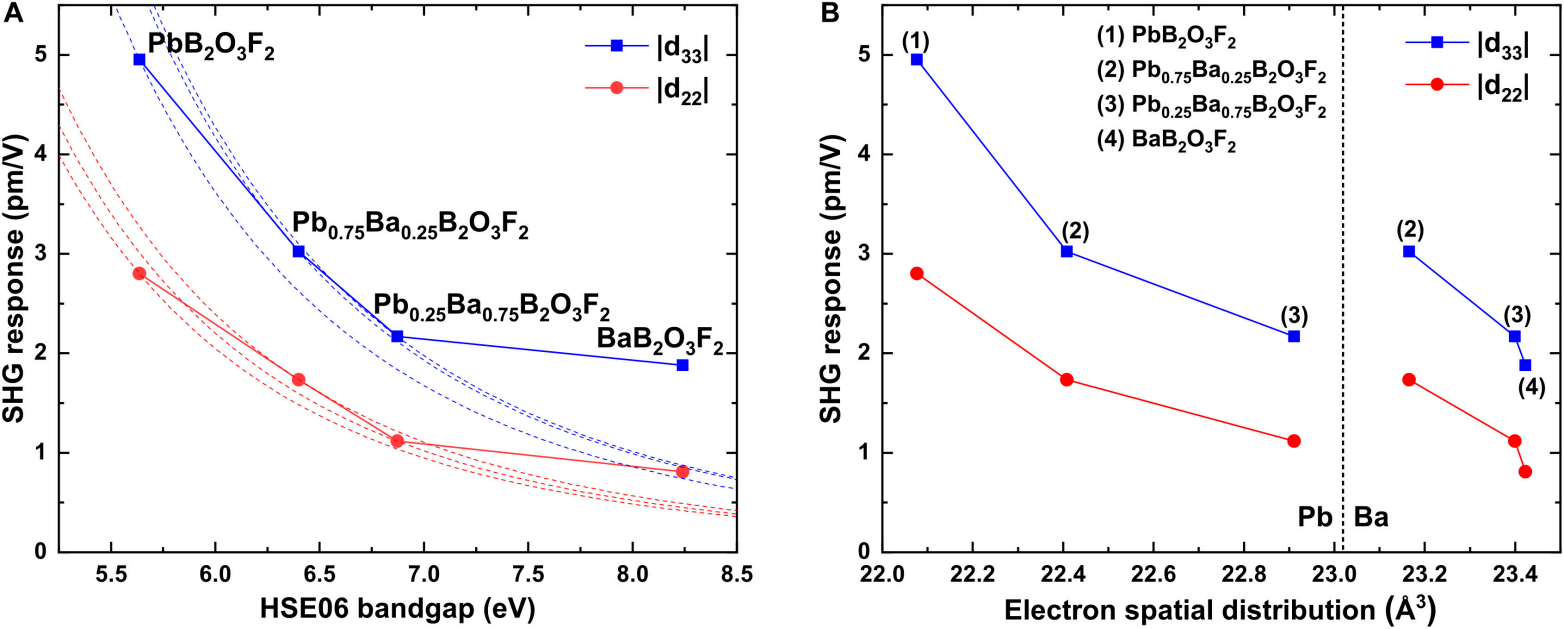
SHG response decreases along with the increase of (A) bandgap and (B) electron spatial distribution (ESD) in MB_2_O_3_F_2_ (M = Pb, Pb_0.75_Ba_0.25_, Pb_0.25_Ba_0.75_, Ba).

On the other hand, to explore the trends of property changing from PbB_2_O_3_F_2_ to BaB_2_O_3_F_2_, two virtual middle structures, Pb_0.75_Ba_0.25_B_2_O_3_F_2_ (Pb_0.75_Ba_0.25_) and Pb_0.25_Ba_0.75_B_2_O_3_F_2_ (Pb_0.25_Ba_0.75_) are introduced. As shown in Fig. 6A, along with the bandgap increasing, the |d_33_| and |d_22_| decrease, as the negative relationship between bandgap and SHG response. Here, we add a trend line with the formula *d* = *a* / *E*_g_^5^, where *d* and *E*_g_ are SHG coefficient and HSE06 bandgap values, and the constant *a* is calculated by the value of *d* and *E*_g_. Of course, this formula only considers the bandgap effect and ignore the momentum matrix elements [71], so it is a very rough approximation to estimate the trends of SHG coefficients along with bandgaps semiquantitatively. Furthermore, as shown in Table S13, a single Pb atom makes much more contributions to *d*_22_ than Ba (22% to 4% in Pb_0.75_Ba_0.25_ and 33% to 7% in Pb_0.25_Ba_0.75_), while Pb and Ba show similar contributions to *d*_33_ (12% to 9% in Pb_0.75_Ba_0.25_ and 13% to 13% in Pb_0.25_Ba_0.75_). Interestingly, the contributions of a single Ba atom develop both in *d*_22_ and *d*_33_ as the proportion of Ba content increases. Therefore, the SHG coefficient trends of PbB_2_O_3_F_2_, Pb_0.75_Ba_0.25,_ and Pb_0.25_Ba_0.75_ do not vary so much, while the SHG coefficients of BaB_2_O_3_F_2_ are much larger than the expected value according to the trend lines.

From Fig. S10, the location of Pb-6p in conduction band minimum is nearer to the Fermi surface than that of Ba-5d for the stereochemical activity of the lone pairs. Meanwhile, the SnB_2_O_3_F_2_ obtains a smaller bandgap than PbB_2_O_3_F_2_ for stronger stereochemical activity. Table 3 also shows that Ba^2+^ contributes the main part of *d*_33_ in BaB_2_O_3_F_2_, so the BaB_2_O_3_F_2_ could maintain a large SHG coefficient when obtaining a large bandgap. Furthermore, we also calculate the ESD value of Pb_0.75_Ba_0.25_ and Pb_0.25_Ba_0.75_ and compared them with PbB_2_O_3_F_2_ and BaB_2_O_3_F_2_. Then, Fig. 6B shows SHG response decreasing along with the increase of ESD in MB_2_O_3_F_2_ (M = Pb, Ba). It is important to note that the above conclusion has only been obtained in the case of cation substitution between Pb and Ba in MB_2_O_3_F_2_ structures.

## Conclusion

Through the investigation of a series of barium borates, Ba_5_(BO_3_)_3_Cl, Ba_3_B_6_O_11_F_2_, Ba_2_B_5_O_9_Cl, Ba_2_BO_3_Br, BaB_5_O_7_F_3_, and BaB_2_O_3_F_2_, along with their prototypes of cation substitutions lead borates, we found that ESD is very useful to describe the electron occupied space, and the magnitudes of ESD are similar in the isostructural substitutions. As the aim of chemical substitutions is to develop performances, three cases, one borate fluoride, and two fluorooxoborates, in 6 cation substitutions from lead to barium have achieved the property optimization, enlarging bandgaps to enter the DUV region (6.2 eV, 200 nm) from outside DUV as the absence of lone pair in metal cations, and maintaining relatively strong SHG responses (> 2 KDP) for the reason that metal cations show less contribution to SHG responses than the anion frames. The cation substitution between Pb and Ba in MB_2_O_3_F_2_ structures shows the negative relation between ESDs and SHG responses, which indicates the direction of searching for the NLO material with larger SHG responses. In conclusion, by analyzing the cation variations, a cation substitution approach toward DUV NLO materials has been proposed, and two predicted fluorooxoborates BaB_5_O_7_F_3_ and BaB_2_O_3_F_2_ have achieved the balance between large bandgaps and strong responses.

## Experimental and Computational Methods

### Experiment details

#### Solid-state synthesis

Polycrystalline samples of Ba_2_B_5_O_9_Cl and Pb_2_B_5_O_9_Cl were prepared by using standard high-temperature solid-state techniques. For Pb_2_B_5_O_9_Cl, a stoichiometric mixture of PbCl_2_ and H_3_BO_3_ was heated at 473 K for 10 h, cooled and ground, and then heated again at 873 K for 48 h. For Ba_2_B_5_O_9_Cl, a stoichiometric mixture of BaCl_2_·2H_2_O, BaCO_3_, Ba(OH)_2_, and H_3_BO_3_ was heated at 573 K for 10 h, cooled and ground, and then heated again at 1153 K for 48 h. The starting materials of PbCl_2_, BaCl_2_·2H_2_O, BaCO_3_, and H_3_BO_3_ were gathered from commercial sources without any purification.

#### Powder X-ray diffraction

XRD patterns of Ba_2_B_5_O_9_Cl and Pb_2_B_5_O_9_Cl were obtained on an automated Bruker D2 X-ray diffractometer equipped with a diffracted beam monochromator set for Cu-Kα radiation (*λ* = 1.5418 Å) at room temperature in the angular range of 2*θ* = 10° to 70° with a scan step of 0.01° and a fixed counting time of 0.1 s/step.

#### UV-visible-near-infrared diffuse reflectance spectrum

The diffuse reflectance spectrum was measured by a Shimadzu SolidSpec-3700DUV spectrophotometer at room temperature from 175 to 2600 nm.

#### Second-order NLO measurements

Powder SHG effects were measured by using the Kurtz-Perry method with a Q-switched Nd: YVO_4_ solid-state laser at 1064 nm [72]. Polycrystalline samples of Ba_2_B_5_O_9_Cl and Pb_2_B_5_O_9_Cl were ground and sieved into the following particle size ranges: 38 to 55, 55 to 88, 88 to 105, 105 to 150, 150 to 200 μm. To make relevant comparisons with known SHG materials, the crystalline KDP sample was also ground and sieved into the same particle size ranges.

### Computation details

#### Electronic structure calculation

In this paper, the CASTEP package, a plane wave pseudo-potential method [73], was used for the DFT calculation of electronic structure properties. With the GGA-PBE functionals were chosen as the exchange-correlation functionals [74]. Norm-conserving pseudopotential [75] was used with the element valance states, Ba 5s^2^5p^6^6s^2^, Pb 5s^2^5p^6^5d^10^6s^2^6p^2^, B 2s^2^2p^1^, O 2s^2^2p^4^, F 2s^2^2p^5^, Cl 3s^2^3p^5^, and Br 4s^2^4p^5^. The cutoff energies of M_2_Ba_3_(BO_3_)_3_Cl, M_3_B_6_O_11_F_2_, M_2_B_5_O_9_Cl, M_2_BO_3_Br, MB_5_O_7_F_3_ and MB_2_O_3_F_2_ (M = Ba^2+^, Pb^2+^) were all no more than 940 eV and the Monkhorst-Pack k-point grids were 3 × 3 × 3, 4 × 3 × 3, 6 × 1 × 4, 4 × 4 × 2, 4 × 4 × 3, and 5 × 5 × 4, respectively.

#### SHG coefficient calculation

When the phase-matching condition is met, the SHG conversion efficiency of an NLO material is significantly determined by the SHG coefficients [76]. Also, the SHG coefficient components are relevant to second-order nonlinear susceptibilities, *d* = *χ*/2. Through the result of the band structure from the CASTEP package, the second-order nonlinear susceptibilities at the limit of zero frequency, *χ*_ijk_^(2)^(0), can be expressed as the sum of the contribution of the virtual-electron processes and the VH processes [19,71].

$$\chi_{\mathrm{ijk}}^{\left(2\right)}=\chi_{\mathrm{ijk}}^{\left(2\right)}\left(\mathrm{VE}\right)+\chi_{\mathrm{ijk}}^{\left(2\right)}\left(\mathrm{VH}\right)$$

χijk2VE=e32ℏm3∑vcc′∫d3k⃑4π3PijkImpvcipcc′jpc′vk1ωcv3ωvc′2+2ωvc4ωc′v

χijk2VH=e32ℏm3∑vv′c∫d3k⃑4π3PijkImpvv′ipv′cjpcvk1ωcv3ωv′c2+2ωvc4ωcv′

Here, *i*, *j*, and *k* are Cartesian components, *v* (*v'*) and *c* (*c'*) are valence and conduction bands, and *P*(*ijk*) represents full permutation.

## Associated Content

### Supplementary Materials

Phonon spectra, powder XRD patterns, total and partial density of states, SHG density, comparison of typical NLO materials in bandgap and SHG response, structure information of predicted structures, and real-space atom-cutting results of SHG coefficients are shown in the Supplementary Materials.

## Data Availability

Data will be made available from the corresponding author upon reasonable request.
