# Supplementary material for: A Cation-Driven Approach toward Deep-Ultraviolet Nonlinear Optical Materials
Source: Research (Wash D C). 2023 Mar 9;6:0053. doi: 10.34133/research.0053 (PMC10013791; doi:10.34133/research.0053)
Supplement: Supplementary Materials — Figs. S1 to S13. Tables S1 to S14. [file research.0053.f1.docx]

**Supporting information**

**A cation-driven approach toward** **deep-ultraviolet nonlinear optical materials**

Cong Hu^†,‡,§^, Meng Cheng^†,‡,§^, Wenqi Jin^†,‡^, Jian Han^†,‡^, Zhihua Yang*^,†,‡^, Shilie Pan*^,†,‡^

^†^*CAS Key Laboratory of Functional Materials and Devices for Special Environments, Xinjiang Technical Institute of Physics & Chemistry, CAS; Xinjiang Key Laboratory of Electronic Information Materials and Devices, 40–1 South Beijing Road, Urumqi 830011, China*

^‡^*Center of Materials Science and Optoelectronics Engineering, University of Chinese Academy of Sciences, Beijing 100049, China*

**Emails: zhyang@ms.xjb.ac.cn, slpan@ms.xjb.ac.cn*

Figure S1. Phonon spectra of BaB_2_O_3_F_2_.

Figure S2. Phonon spectra of BaB_5_O_7_F_3_.

Figure S3. Powder XRD patterns of Pb_2_B_5_O_9_Cl and Ba_2_B_5_O_9_Cl.

Figure S4. Total and partial density of states of M_2_B_5_O_9_Cl (M = Pb, Ba).

Figure S5. Total and partial density of states of M_2_BO_3_Br (M = Pb, Ba).

Figure S6. Total and partial density of states of M_3_B_6_O_11_F_2_ (M = Pb, Ba).

Figure S7. Total and partial density of states of Pb_2_Ba_3_(BO_3_)_3_Cl and Ba_5_(BO_3_)_3_Cl.

Figure S8. Total and partial density of states of MB_2_O_3_F_2_ (M = Pb, Sn, Ba).

Figure S9. Total and partial density of states of MB_5_O_7_F_3_ (M = Pb, Ba).

Figure S10. SHG densities of Ba_2_BO_3_Br.

Figure S11. SHG densities of BaB_2_O_3_F_2_.

Figure S12. SHG densities of BaB_5_O_7_F_3_.

Figure S13. Total and partial density of states of Pb_0.75_Ba_0.25_B_2_O_3_F_2_ and Pb_0.25_Ba_0.75_B_2_O_3_F_2_.

Table S1. Comparison of typical NLO materials on bandgap and SHG response.

Table S2. Lattice parameters of experimental *P*321 Pb_2_BO_3_Br and predicted *P*321 Ba_2_BO_3_Br.

Table S3. Lattice parameters of experimental *P*31*m* PbB_2_O_3_F_2_ and *P*2_1_ BaB_2_O_3_F_2_, and predicted *P*2_1_ PbB_2_O_3_F_2_ and *P*31*m* BaB_2_O_3_F_2_.

Table S4. Lattice parameters of experimental *Cmc*2_1_ BaB_5_O_7_F_3_ and predicted *Cmc*2_1_ BaB_5_O_7_F_3_.

Table S5. Atomic coordinates, equivalent isotropic displacement parameters for Ba_2_BO_3_Br.

Table S6. Selected bond distances and angles for Ba_2_BO_3_Br.

Table S7. Atomic coordinates, equivalent isotropic displacement parameters for BaB_2_O_3_F_2_.

Table S8. Selected bond distances and angles for BaB_2_O_3_F_2_.

Table S9. Atomic coordinates, equivalent isotropic displacement parameters for BaB_5_O_7_F_3_.

Table S10. Selected bond distances and angles for BaB_5_O_7_F_3_.

Table S11. Atomic coordinates, equivalent isotropic displacement parameters for PbB_2_O_3_F_2_.

Table S12. Selected bond distances and angles for PbB_2_O_3_F_2_.

Table S13. Real-space atom-cutting results of SHG coefficients of MB_2_O_3_F_2_ (M = Pb, Pb_0.75_Ba_0.25_, Pb_0.25_Ba_0.75_, Ba).

Table S14. Real-space atom-cutting results of SHG coefficients of MB_2_O_3_F_2_ (M = Sn, Sn_0.75_Ba_0.25_, Sn_0.25_Ba_0.75_, Ba).


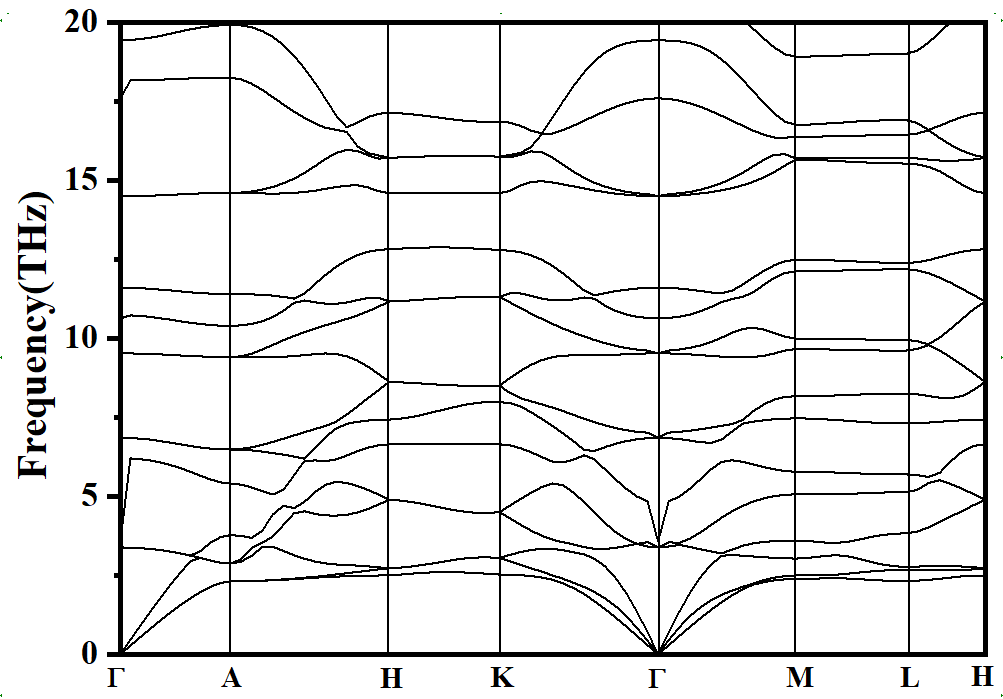


**Figure S1.** Phonon spectra of BaB_2_O_3_F_2_.


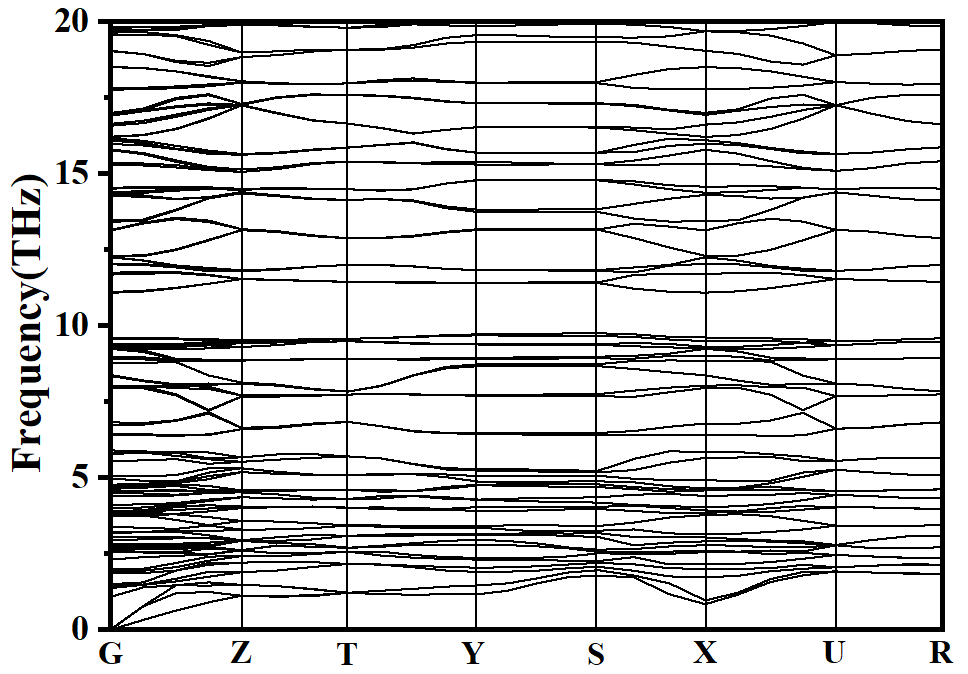


**Figure S2.** Phonon spectra of BaB_5_O_7_F_3_.


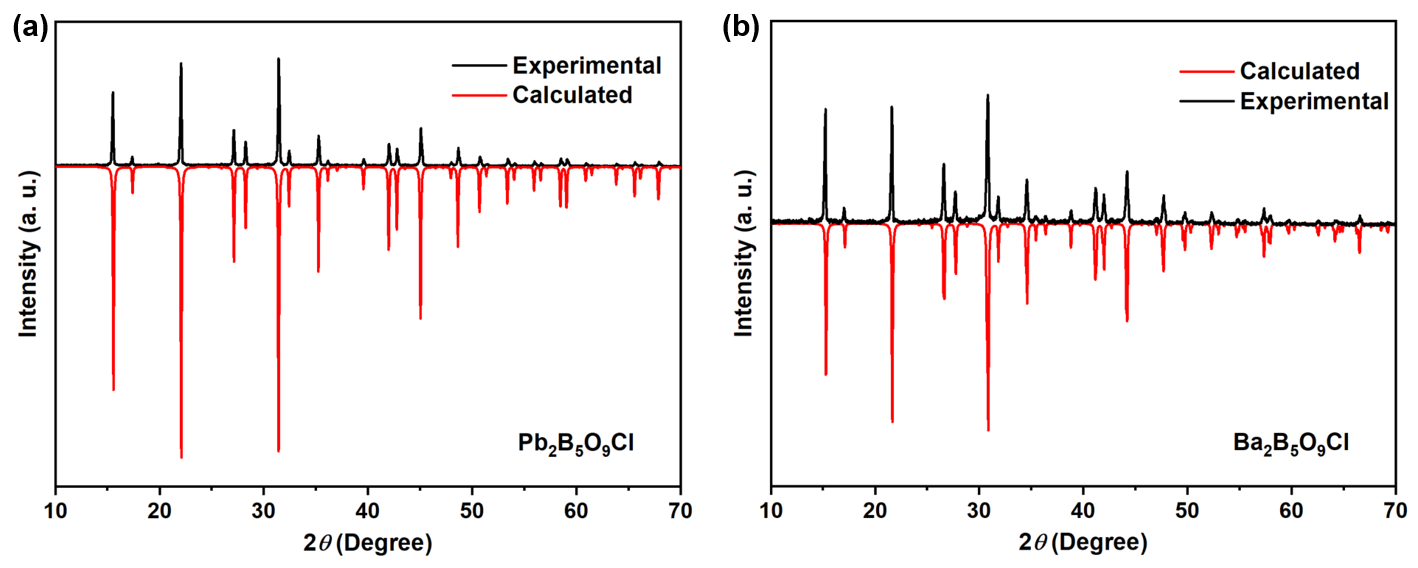


**Figure S3.** Powder XRD patterns of Pb_2_B_5_O_9_Cl (a) and Ba_2_B_5_O_9_Cl (b).


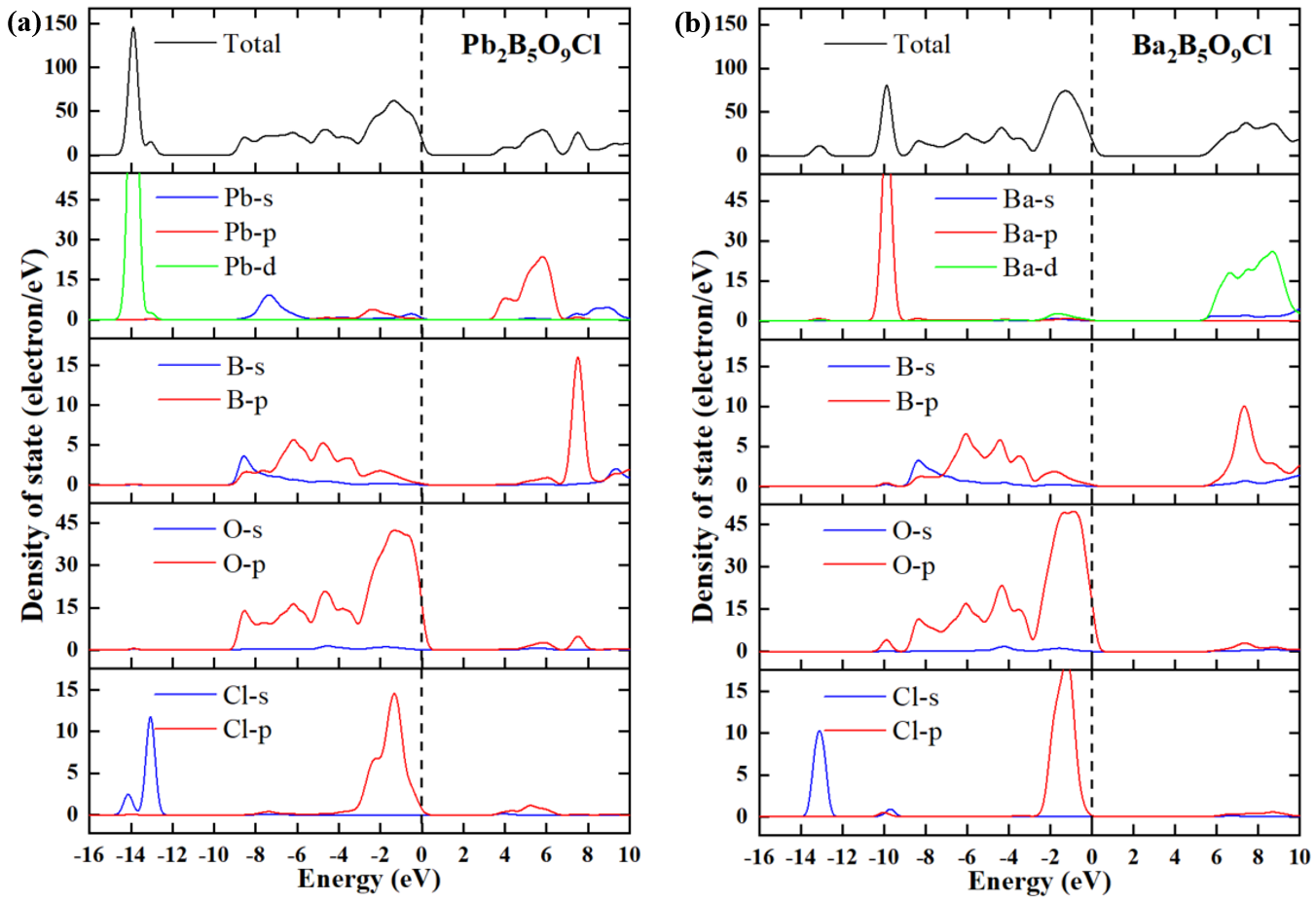


**Figure S4.** Total and partial density of states of M_2_B_5_O_9_Cl (M = Pb, Ba).


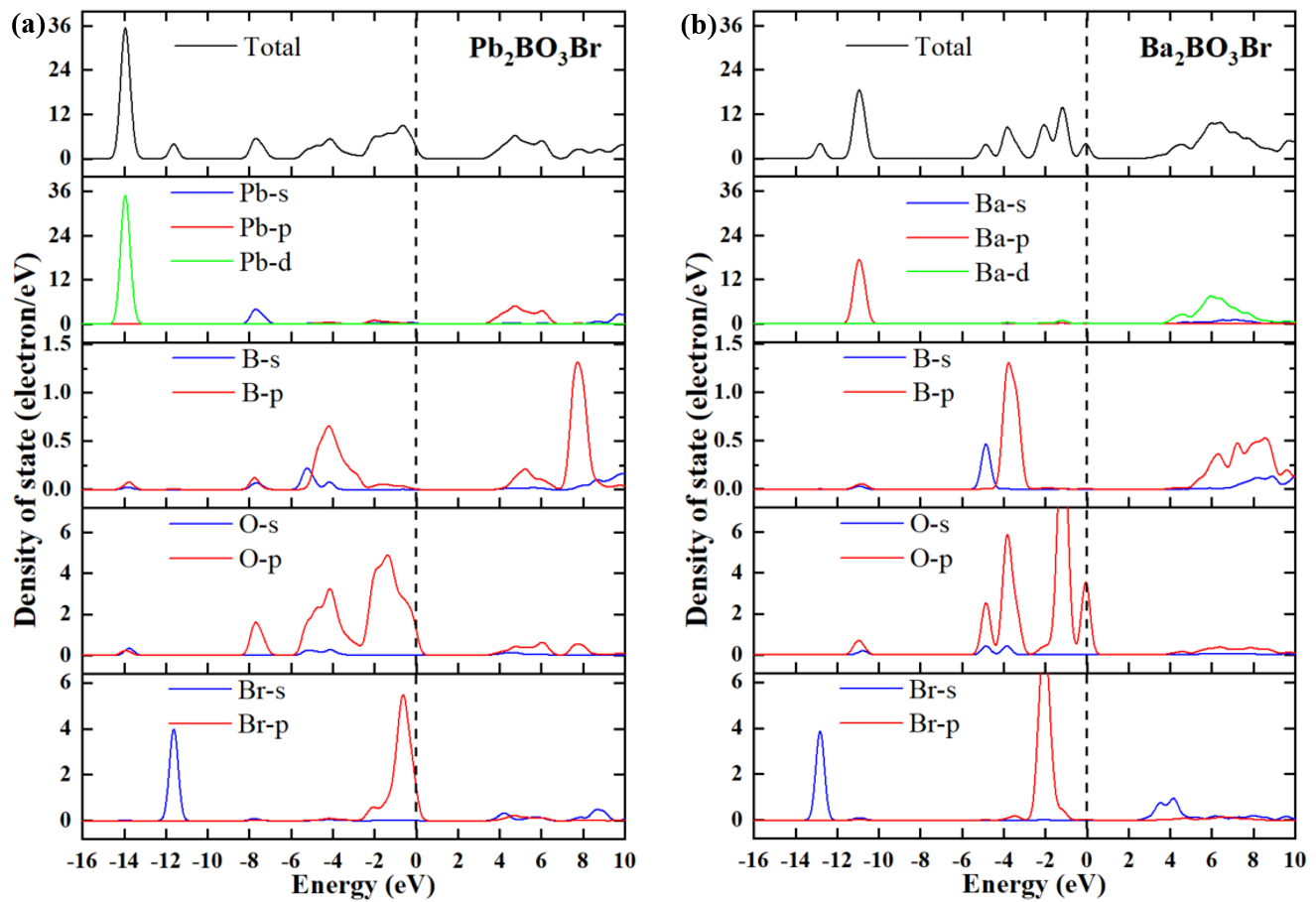


**Figure S5.** Total and partial density of states of M_2_BO_3_Br (M = Pb, Ba).


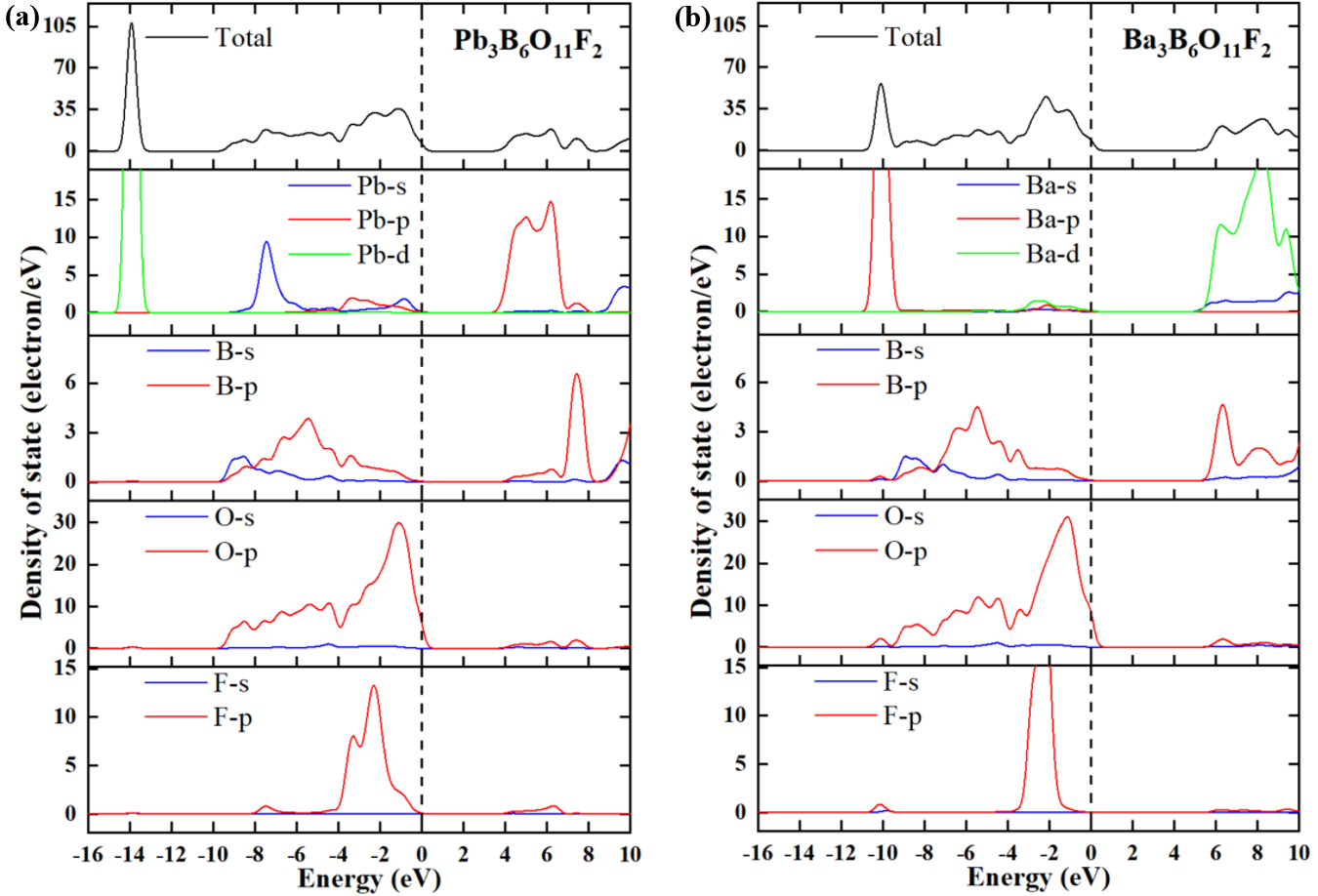


**Figure S6.** Total and partial density of states of M_3_B_6_O_11_F_2_ (M = Pb, Ba).


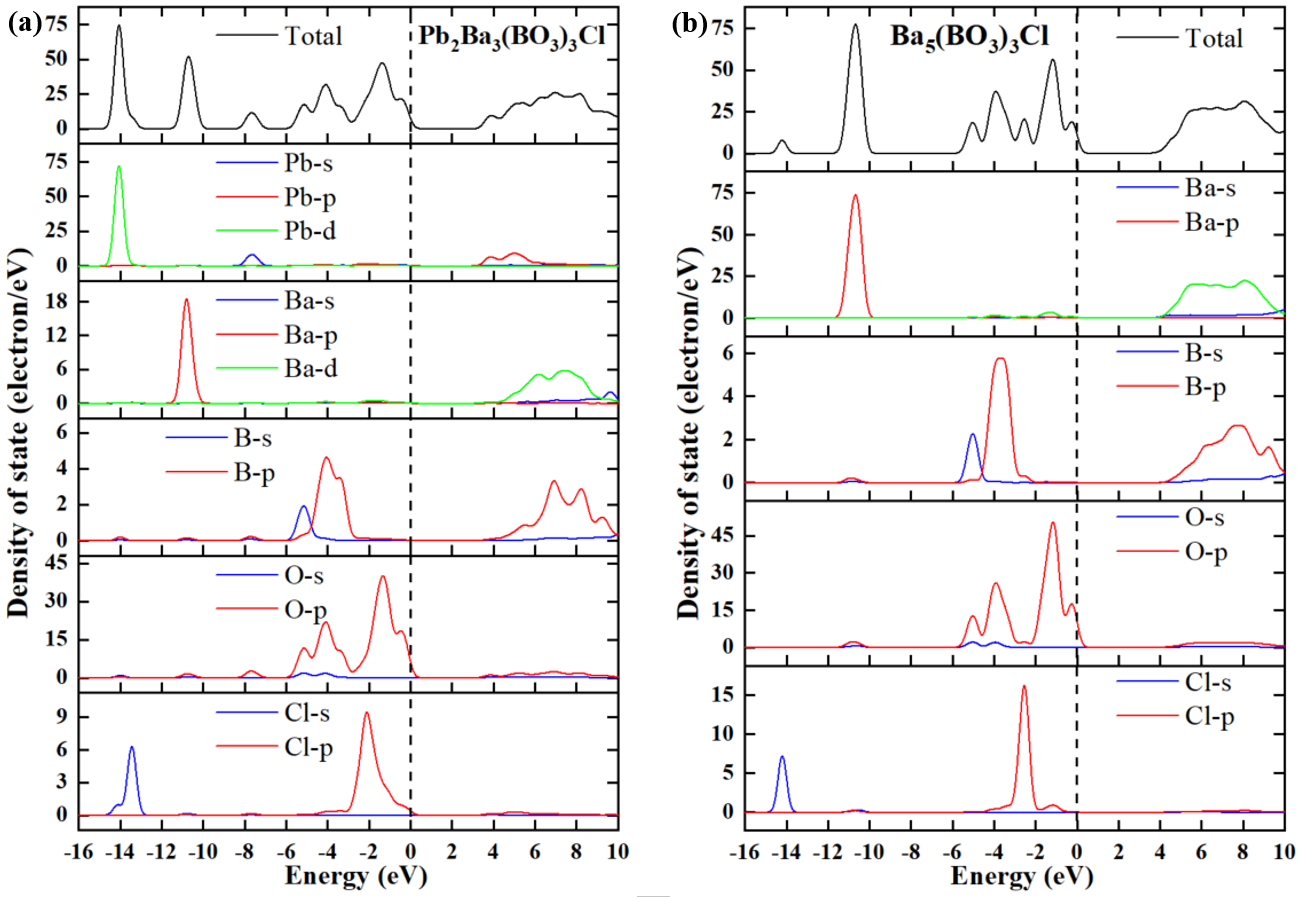


**Figure S7.** Total and partial density of states of Pb_2_Ba_3_(BO_3_)_3_Cl and Ba_5_(BO_3_)_3_Cl.


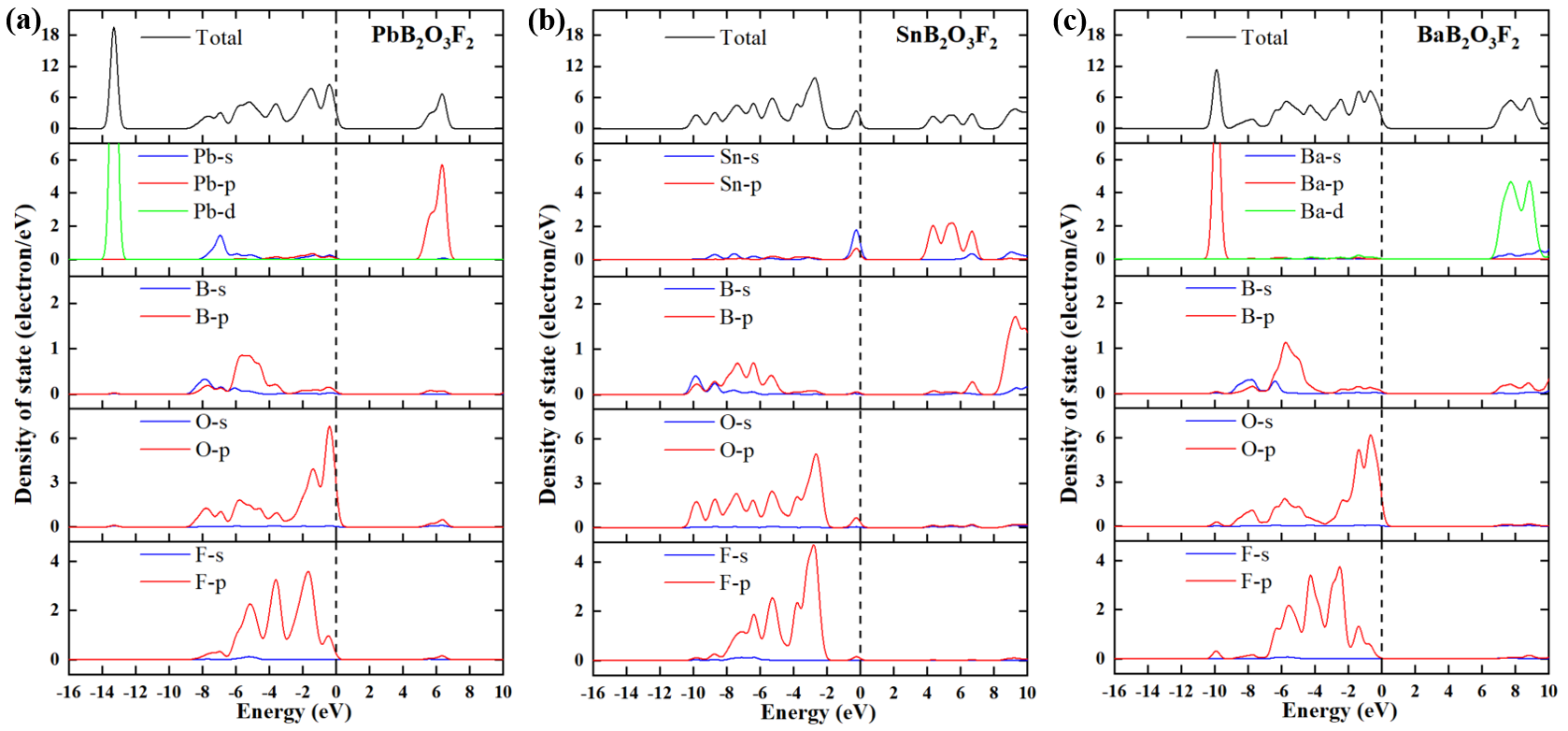


**Figure S8.** Total and partial density of states of MB_2_O_3_F_2_ (M = Pb, Sn, Ba).


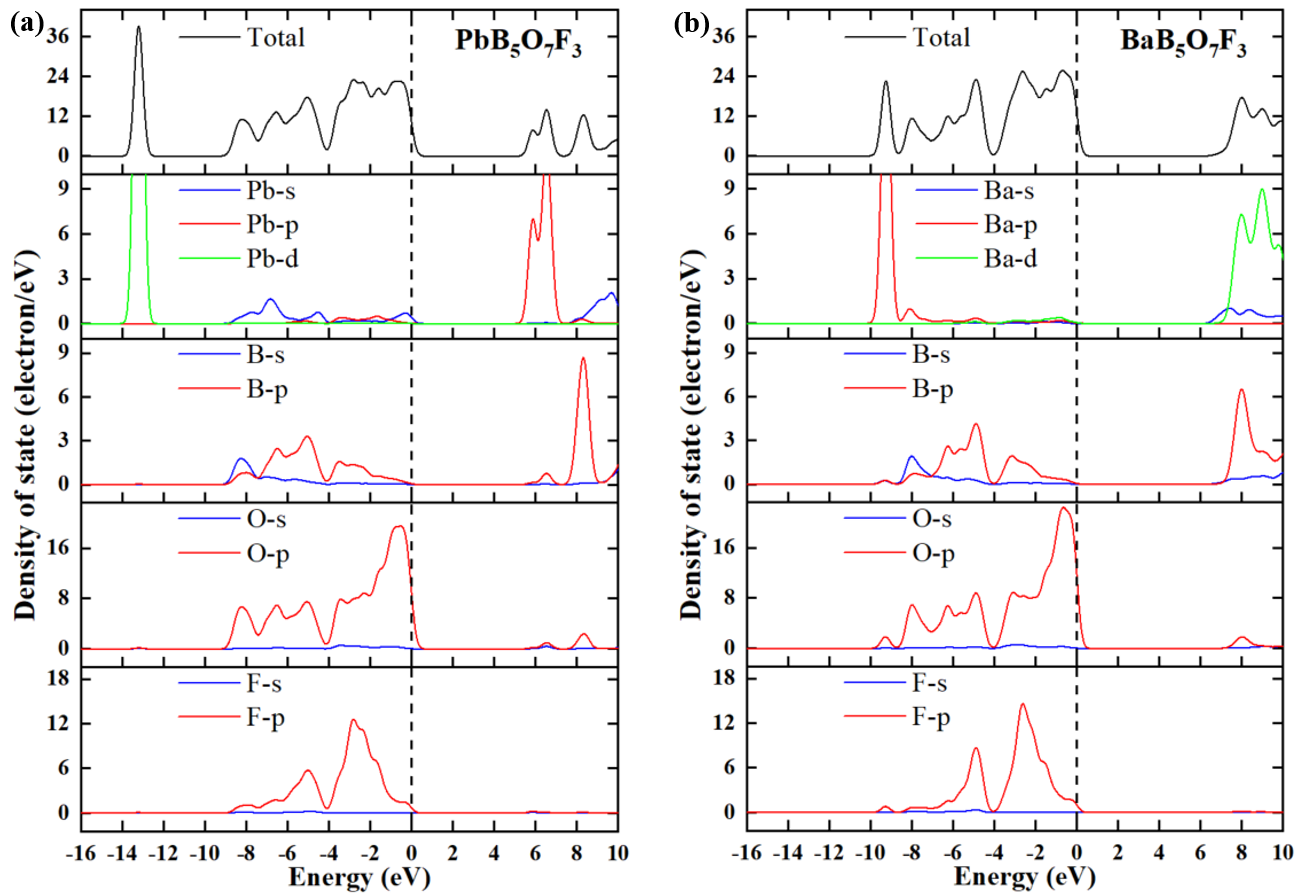


**Figure S9.** Total and partial density of states of MB_5_O_7_F_3_ (M = Pb, Ba).


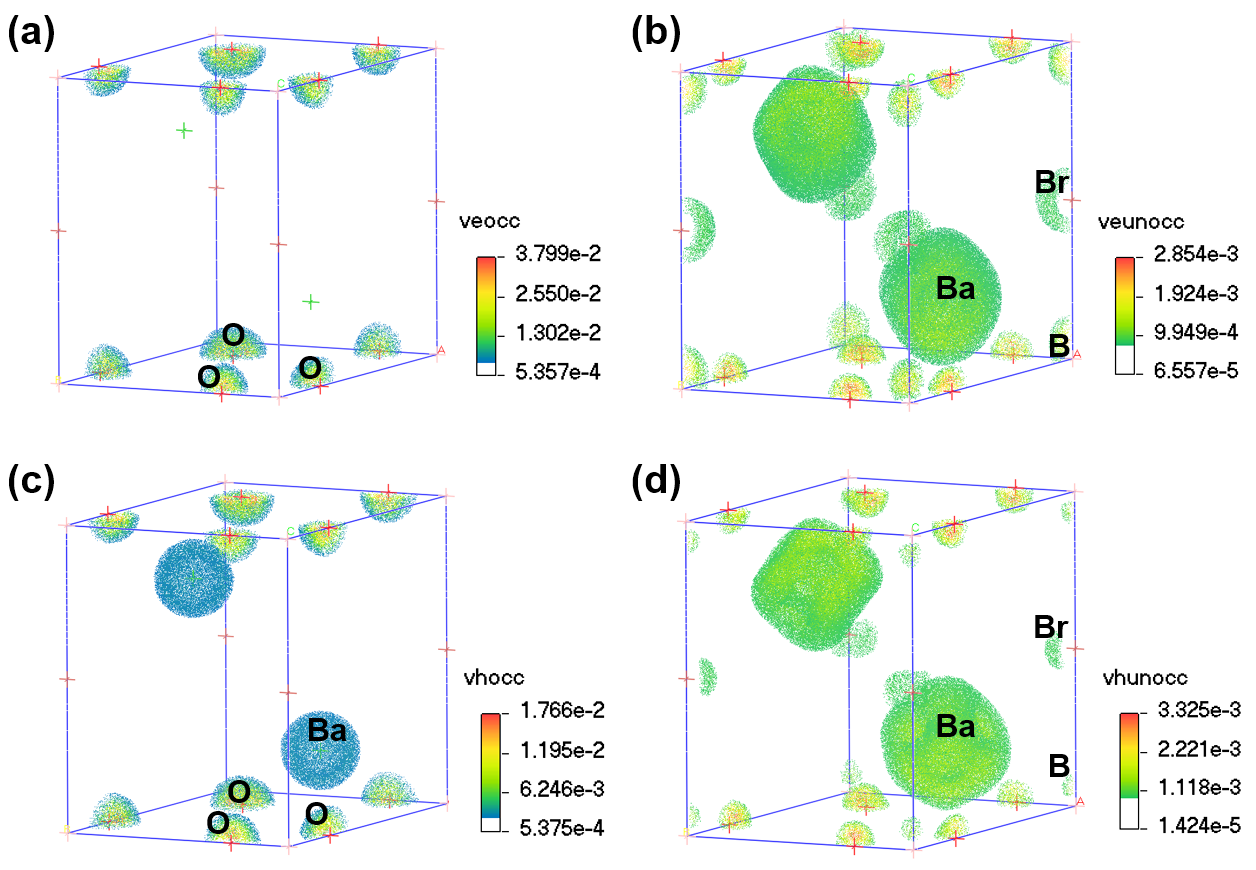


**Figure S10.** SHG densities of Ba_2_BO_3_Br. The SHG coefficient is the sum of the contributions by virtual-electron (VE) processes (a, b) and virtual hole (VH) processes (c, d) from occupied states (a, c) and unoccupied states (b, d).


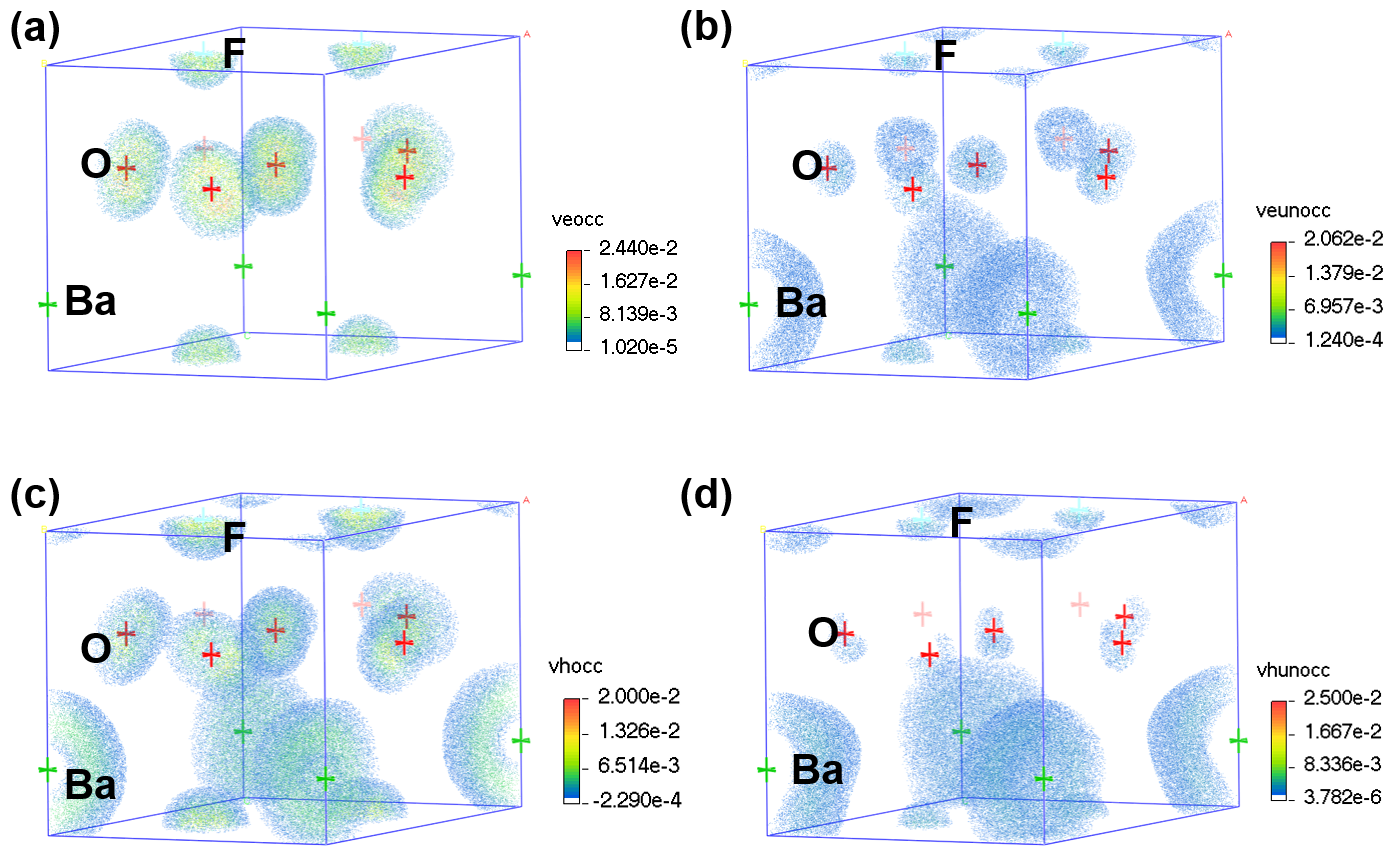


**Figure S11.** SHG densities of BaB_2_O_3_F_2_. The SHG coefficient is the sum of the contributions by virtual-electron (VE) processes (a, b) and virtual hole (VH) processes (c, d) from occupied states (a, c) and unoccupied states (b, d).


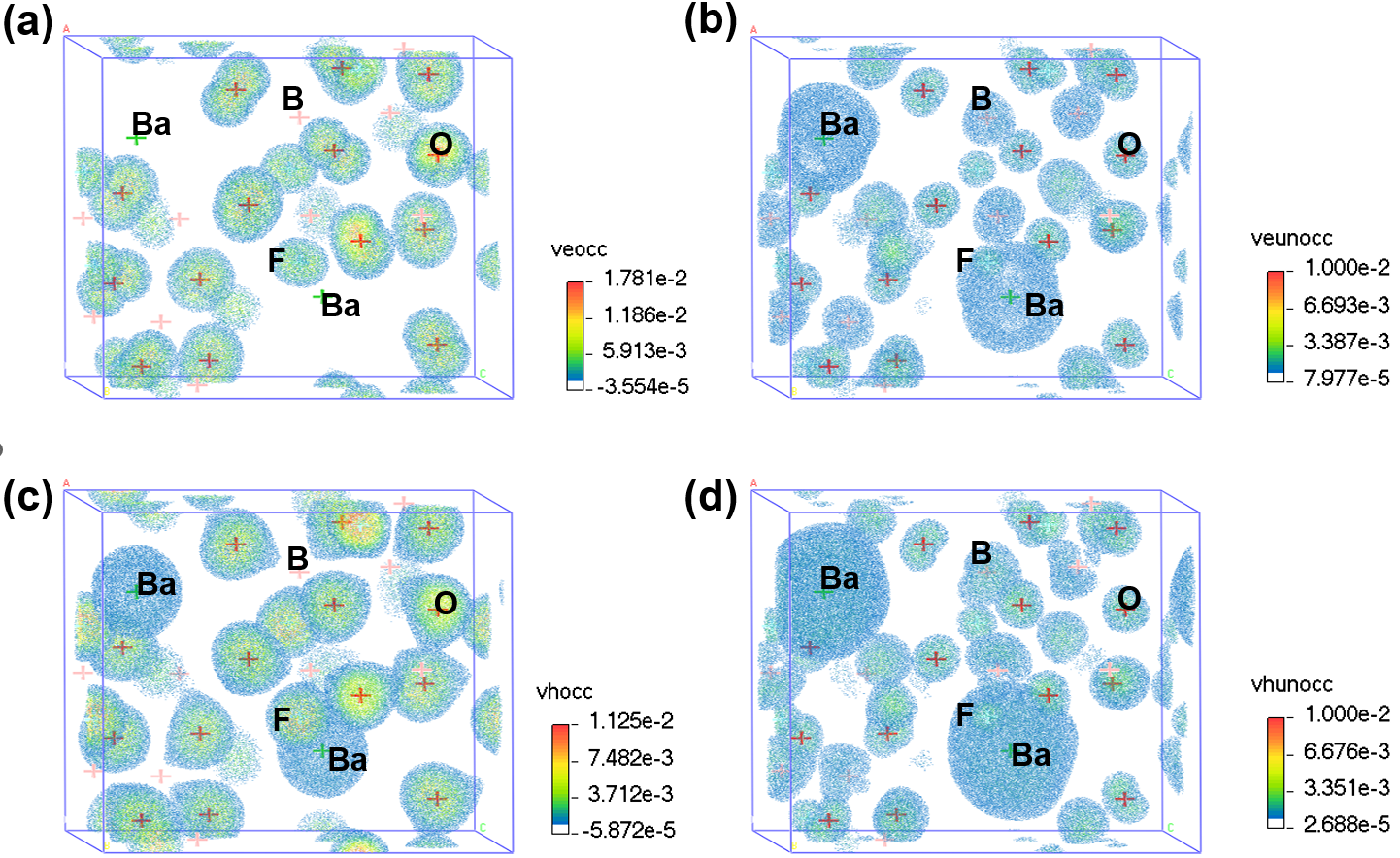


**Figure S12.** SHG densities of BaB_5_O_7_F_3_. The SHG coefficient is the sum of the contributions by virtual-electron (VE) processes (a, b) and virtual hole (VH) processes (c, d) from occupied states (a, c) and unoccupied states (b, d).


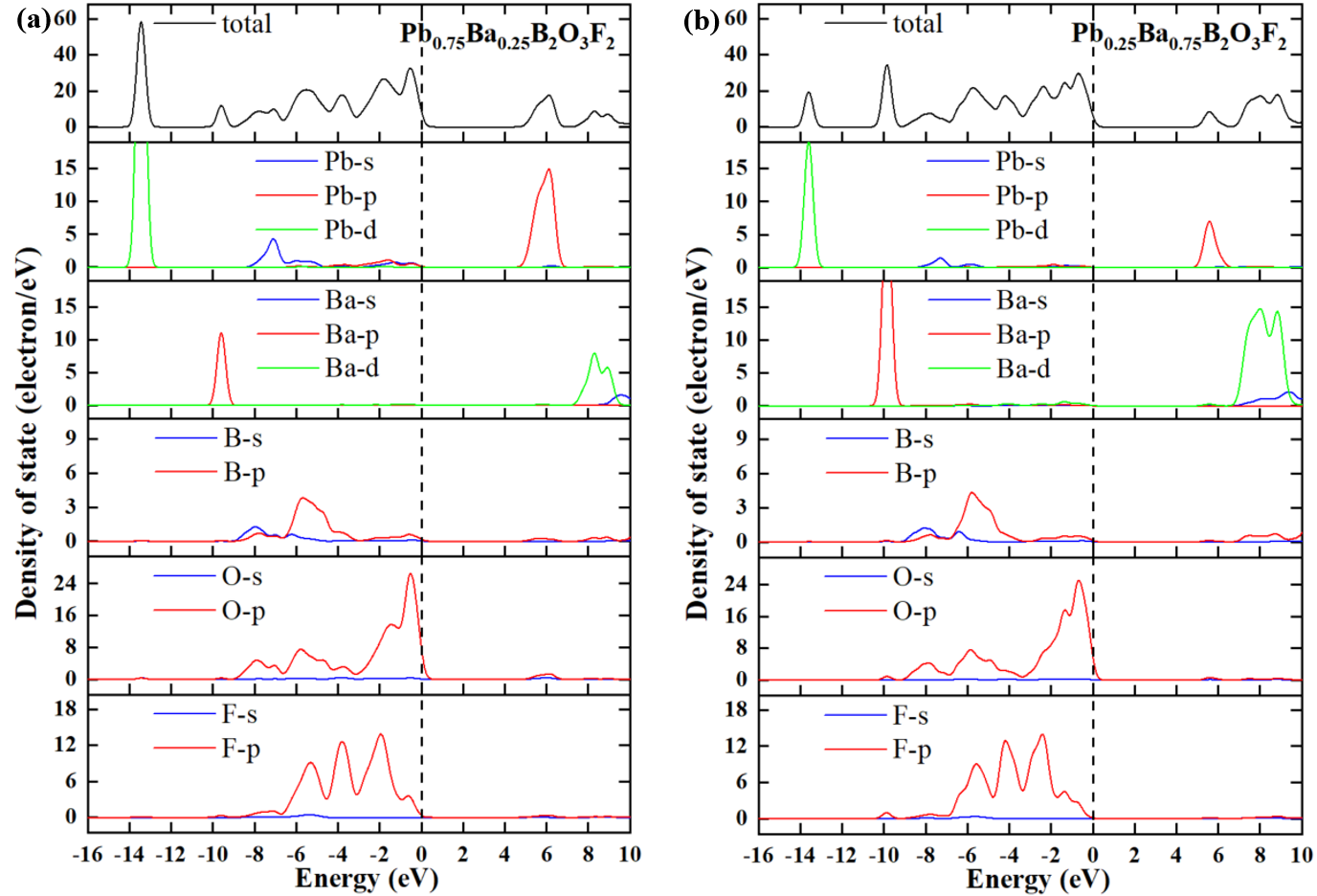


**Figure S13.** Total and partial density of states of Pb_0.75_Ba_0.25_B_2_O_3_F_2_ and Pb_0.25_Ba_0.75_B_2_O_3_F_2_.

**Table S1.** Comparison of typical NLO materials on bandgap and SHG response.

| No. | Compound | Bandgap  (eV) | UV cutoff edge  (nm) | SHG response  (KDP) | Reference |
| --- | --- | --- | --- | --- | --- |
| 1 | Pb_2_B_5_O_9_I | 3.10 | 400 | 13.5 | Ref.^1^ |
| 2 | Cd_4_BiO(BO_3_)_3_ | 3.16 | 392 | 6.0 | Ref.^2^ |
| 3 | Bi_3_TeBO_9_ | 3.22 | 385 | 20.0 | Ref.^3^ |
| 4 | Pb_2_BO_3_Br | 3.33 | 372 | 9.5 | Ref.^4^ |
| 5 | Pb_2_(BO_3_)(NO_3_) | 3.65 | 340 | 9.0 | Ref.^5^ |
| 6 | Pb_2_BO_3_I | 3.76 | 330 | 10.0 | Ref.^6^ |
| 7 | Pb_2_Ba_3_(BO_3_)_3_Cl | 3.97 | 312 | 3.2 | Ref.^7^ |
| 8 | Pb_2_BO_3_Cl | 3.99 | 311 | 9.0 | Ref.^8^ |
| 9 | CsPbCO_3_F | 4.15 | 299 | 13.4 | Ref.^9^ |
| 10 | *α*-BiB_3_O_6_ | 4.33 | 286 | 8.2 | Ref.^10^ |
| 11 | Pb_4_O(BO_3_)_2_ | 4.43 | 280 | 3.0 | Ref.^11^ |
| 12 | BiB_2_O_4_F | 4.43 | 280 | 12.0 | Ref.^12^ |
| 13 | SnB_2_O_3_F_2_ | 4.96 | 250 | 4.0 | Ref.^13^ |
| 14 | PbB_4_O_7_ | 5.17 | 240 | 1.7 | Ref.^14^ |
| 15 | Pb_3_B_6_O_11_F_2_ | 5.17 | 240 | 4.0 | Ref.^15^ |
| 16 | KCdCO_3_F | 5.30 | 234 | 4.6 | Ref.^16^ |
| 17 | PbB_2_O_3_F_2_ | 5.64 | 220 | 13.0 | Ref.^13^ |
| 18 | Cs_3_Zn_6_B_9_O_21_ | 6.20 | 200 | 3.3 | Ref.^17^ |
| 19 | K_7_BaY_2_(B_5_O_10_)_3_ | 6.53 | 190 | 1.2 | Ref.^18^ |
| 20 | Ba_4_B_11_O_20_F | 6.53 | 190 | 4.0 | Ref.^19^ |
| 21 | Ba_3_B_6_O_11_F_2_ | 6.55 | 189 | 3.0 | Ref.^20^ |
| 22 | *β*-BaB_2_O_4_ | 6.56 | 189 | 5.7 | Ref.^21^ |
| 23 | Li_4_Sr(BO_3_)_2_ | 6.67 | 186 | 2.0 | Ref.^22^ |
| 24 | RbNaMgP_2_O_7_ | 6.70 | 185 | 0.9 | Ref.^23^ |
| 25 | Ba_3_Mg_3_(BO_3_)_3_F_3_ | 6.74 | 184 | 1.3 | Ref.^24^ |
| 26 | K_3_B_6_O_10_Cl | 6.89 | 180 | 4.0 | Ref.^25^ |
| 27 | CsLiB_6_O_10_ | 6.89 | 180 | 2.2 | Ref.^26^ |
| 28 | K_2_Al_2_B_2_O_7_ | 6.89 | 180 | 1.0 | Ref.^27^ |
| 29 | CsB_3_O_5_ | 7.43 | 167 | 2.7 | Ref.^28^ |
| 30 | RbBa_2_(PO_3_)_5_ | 7.61 | 163 | 1.4 | Ref.^29^ |
| 31 | LiB_3_O_5_ | 7.75 | 160 | 2.7 | Ref.^30^ |
| 32 | CsKB_8_O_12_F_2_ | 7.76 | 160 | 1.9 | Ref.^31^ |
| 33 | NH_4_B_4_O_6_F | 7.95 | 156 | 3.0 | Ref.^32^ |
| 34 | CsB_4_O_6_F | 8.00 | 155 | 1.9 | Ref.^33^ |
| 35 | NH_4_Be_2_BO_3_F_2_ | 8.10 | 153 | 1.2 | Ref.^34^ |
| 36 | KBe_2_BO_3_F_2_ | 8.44 | 147 | 1.2 | Ref.^35^ |

**Table S2.** Lattice parameters of experimental *P*321 Pb_2_BO_3_Br and predicted *P*321 Ba_2_BO_3_Br.

|  | Pb_2_BO_3_Br^4^ | Pb_2_BO_3_Br  (opt.)^a^ | predicted Ba_2_BO_3_Br  (opt.) |
| --- | --- | --- | --- |
| Z | 1 | 1 | 1 |
| a (Å) | 4.961 | 5.026 | 5.295 |
| b (Å) | 4.961 | 5.026 | 5.295 |
| c (Å) | 6.735 | 6.811 | 7.399 |
| V (Å^3^) | 143.563 | 149.003 | 179.522 |

^a^ opt. (optimization) is referring to the structure after geometric structure optimizations by DFT calculation

**Table S3.** Lattice parameters of experimental *P*31*m* PbB_2_O_3_F_2_ and *P*2_1_ BaB_2_O_3_F_2_, and predicted *P*2_1_ PbB_2_O_3_F_2_ and *P*31*m* BaB_2_O_3_F_2_.

|  | PbB_2_O_3_F_2_^13^  (*P*31*m*) | PbB_2_O_3_F_2_  (*P*31*m*, opt.) | predicted BaB_2_O_3_F_2_  (*P*31*m*, opt.) | BaB_2_O_3_F_2_^36^  (*P*2_1_) | BaB_2_O_3_F_2_  (*P*2_1_, opt.) | predicted PbB_2_O_3_F_2_  (*P*2_1_, opt.) |
| --- | --- | --- | --- | --- | --- | --- |
| Z | 1 | 1 | 1 | 2 | 2 | 2 |
| a (Å) | 4.521 | 4.557 | 4.583 | 4.455 | 4.469 | 4.424 |
| b (Å) | 4.521 | 4.557 | 4.583 | 4.265 | 4.281 | 4.239 |
| c (Å) | 4.706 | 4.851 | 4.940 | 9.239 | 9.402 | 8.926 |
| β (^o^) |  |  |  | 91.104 | 91.392 | 90.748 |
| V (Å^3^) | 83.305 | 87.235 | 89.867 | 175.532 | 179.824 | 167.378 |

**Table S4.** Lattice parameters of experimental *Cmc*2_1_ BaB_5_O_7_F_3_ and predicted *Cmc*2_1_ BaB_5_O_7_F_3_.

|  | PbB_5_O_7_F_3_^37^ | PbB_5_O_7_F_3_ (opt.) | BaB_5_O_7_F_3_ (opt.) |
| --- | --- | --- | --- |
| Z | 4 | 1 | 1 |
| a (Å) | 10.025 | 10.158 | 10.304 |
| b (Å) | 8.764 | 8.937 | 9.186 |
| c (Å) | 8.073 | 8.283 | 8.425 |
| V (Å^3^) | 709.287 | 749.176 | 797.482 |

**Table S5.** Atomic coordinates (×10^4^), equivalent isotropic displacement parameters (Å^2^×10^3^) for Ba_2_BO_3_Br. U(eq) is defined as one third of the trace of the orthogonalized U_ij_ tensor.

| Atom | *x* | *y* | *z* | *U(eq)* |
| --- | --- | --- | --- | --- |
| Ba(1) | 3333.3 | 6666.7 | 7960.3 | 14.82 |
| B(1) | 0 | 10000 | 10000 | 0.04 |
| O(1) | 2608.9 | 10000 | 10000 | 0.27 |
| Br(1) | 0 | 10000 | 5000 | 33.96 |

**Table S6.** Selected bond distances (Å) and angles (deg) for Ba_2_BO_3_Br.

| Bond | Distances (Å) |
| --- | --- |
| B (1) - O(1) | 1.3813 |

| Bond | Angels (deg) |
| --- | --- |
| O(1)- B (1)-O(1)#1 | 120.00 |
| O(1) #1- B (1)-O(1)#2 | 120.00 |
| O(1)- B (1)-O(1)#2 | 120.00 |

Symmetry transformations used to generate equivalent atoms:

#1 1-y, 2+x-y, z #2 1-x+y, 1-x, z

**Table S7.** Atomic coordinates (×10^4^), equivalent isotropic displacement parameters (Å^2^×10^3^) for BaB_2_O_3_F_2_. U(eq) is defined as one third of the trace of the orthogonalized U_ij_ tensor.

| Atom | *x* | *y* | *z* | *U(eq)* |
| --- | --- | --- | --- | --- |
| Ba(1) | 10000 | 0 | -2165.8 | 5.2 |
| B(1) | 13333.3 | -3333.3 | -6959.7 | 1.77 |
| O(1) | 10000 | -4075.7 | -6123.1 | 1.24 |
| F(1) | 13333.3 | -3333.3 | -10066.3 | 5.2 |

**Table S8.** Selected bond distances (Å) and angles (deg) for BaB_2_O_3_F_2_.

| Bond | Distances (Å) |
| --- | --- |
| Ba (1) - O(1) | 2.710 |
| Ba (1) - O(1)#1 | 2.710 |
| Ba (1) - O(1) | 2.710 |
| Ba (1) - O(1) | 2.710 |
| B (1)#3 – O(1) | 1.450 |
| B (1) – O(1) | 1.450 |
| B (1) – O(1)#4 | 1.450 |
| B (1) – O(1)#5 | 1.450 |
| B (1) – F(1) | 1.541 |

| Bond | Angels (deg) |
| --- | --- |
| O(1)- B (1)-O(1) | 112.1633 |
| O(1)#1- B (1)-F(1) | 106.6227 |

Symmetry transformations used to generate equivalent atoms:

| #1 2+y-x,1-x,+z; | #2 1-y,-1+x-y,+z; | #3 1+Y,-2+X,+Z; |
| --- | --- | --- |
| #4 1-y,-2+x-y,+z; | #5 3+y-x,1-x,+z |  |

**Table S9.** Atomic coordinates (×10^4^), equivalent isotropic displacement parameters (Å^2^×10^3^) for BaB_5_O_7_F_3_. U(eq) is defined as one third of the trace of the orthogonalized U_ij_ tensor.

| Atom | *x* | *y* | *z* | *U(eq)* |
| --- | --- | --- | --- | --- |
| Ba(1) | 5000 | -7501.9 | -6058.3 | 10.97 |
| B(1) | 3645.5 | -11585 | -5257.9 | 12.39 |
| B(2) | 2710.1 | -12675.3 | -7793.2 | 11.61 |
| B(3) | 5000 | -3294.3 | -7182.5 | 13.38 |
| O(1) | 2613.3 | -11972.2 | -6355.2 | 10.15 |
| O(2) | 6583.6 | -7805.6 | -8674.3 | 16.41 |
| O(3) | 3880.2 | -3229.9 | -8240.3 | 12.71 |
| O(4) | 5000 | -2090.1 | -5925.4 | 6.11 |
| F(1) | 3707.7 | -10048.1 | -5146.9 | 21.51 |
| F(2) | 5000 | -4625.8 | -6322.4 | 24.9 |

**Table S10.** Selected bond distances (Å) and angles (deg) for BaB_5_O_7_F_3_.

| Bond | Distances (Å) | Bond | Distances (Å) |
| --- | --- | --- | --- |
| Ba(1)#3 – O(1) | 2.738 | B (1) - O(1) | 1.446 |
| Ba(1) – O(2) | 2.738 | B (1) - O(2)#1 | 1.457 |
| Ba(1)#7 – O(3) | 2.705 | B (1) - F(1) | 1.424 |
| Ba(1) – O(1)#5 | 2.738 | B (1) - O(4)#2 | 1.569 |
| Ba(1) – O(1)#8 | 2.738 | B (1)#4 - O(2) | 1.457 |
| Ba(1) – O(2)#9 | 2.738 | B (1)#12 - O(4) | 1.569 |
| Ba(1) – O(3)#10 | 2.705 | B (1)#6 - O(4) | 1.569 |
| Ba(1) – O(3)#11 | 2.705 | B (2) – O(1) | 1.369 |
| Ba(1) – F(1) | 2.805 | B (2) – O(2)#3 | 1.376 |
| Ba(1) – F(1)#9 | 2.805 | B (2) – O(3)#2 | 1.358 |
| Ba(1) – F(2) | 2.665 | B (2)#5 – O(2) | 1.5175 |
| B (3) – O(3)#9 | 1.451 | B (2)#6 – O(3) | 1.358 |
| B (3) – O(4) | 1.529 | B (3) – O(3) | 1.451 |
| B (3) – F(2) | 1.424 |  |  |

| Bond | Angels (deg) | Bond | Angels (deg) |
| --- | --- | --- | --- |
| F(1)- B (1)-O(1) | 108.70 | O(2)#3 - B (2)-O(3)#2 | 124.27 |
| F(1) - B (1)-O(4)#2 | 106.25 | F(2) - B (3)-O(3) | 110.04 |
| F(1) - B (1)-O(2)#1 | 109.46 | F(2) - B (3)-O(4) | 106.34 |
| O(1) - B (1)-O(4)#2 | 110.52 | F(2) - B (3)-O(3)#9 | 110.04 |
| O(1) - B (1)-O(2)#1 | 111.22 | O(3) - B (3)-O(4) | 112.85 |
| O(2)#1 - B (1)-O(4)#2 | 110.54 | O(3) - B (3)-O(3)#9 | 104.77 |
| O(1) - B (2)-O(3)#2 | 118.97 | O(3)#9 - B (3)-O(4) | 112.85 |
| O(1) - B (2)-O(2)#3 | 116.73 |  |  |

Symmetry transformations used to generate equivalent atoms:

| #1 1-x,-2-y,1/2+z | #2 x,-1+y,z | #3 -1/2+x,-1/2+y,z |
| --- | --- | --- |
| #4 1-x,-2-y,-1/2+z  #7 1-x,-1-y,-1/2+z | #5 1/2+x,1/2+y,z  #8 1/2-x,1/2+y,z | #6 x,1+y,z  #9 1-x,y,z |
| #10 1-x,-1-y,1/2+z | #11 x,-1-y,1/2+z | #12 1-x,1+y,z |

**Table S11.** Atomic coordinates (×10^4^), equivalent isotropic displacement parameters (Å^2^×10^3^) for PbB_2_O_3_F_2_. U(eq) is defined as one third of the trace of the orthogonalized U_ij_ tensor.

| Atom | *x* | *y* | *z* | *U(eq)* |
| --- | --- | --- | --- | --- |
| Pb(1) | 7440.8 | 8075.4 | 8425 | 12.7 |
| B(1) | 1732.3 | 3109.7 | 6445.9 | 12.7 |
| B(3) | 3266 | 7830.9 | 5024.8 | 12.7 |
| O(1) | 2350.5 | 6419.8 | 6388 | 12.7 |
| O(3) | 6322.8 | 6400 | 4670.5 | 12.7 |
| O(5) | 8604.1 | 2305.2 | 6285.5 | 12.7 |
| F(1) | 2427.6 | 7604.9 | 20.4 | 12.7 |
| F(3) | 2653.9 | 2061.1 | 7932.2 | 12.7 |

**Table S12.** Selected bond distances (Å) and angles (deg) for PbB_2_O_3_F_2_.

| Bond | Distances (Å) | Bond | Distances (Å) |
| --- | --- | --- | --- |
| Pb (1) - F(2) | 2.7170 | B(1) – F(3) | 1.4618 |
| Pb (1) - F(1)#6 | 2.6807 | B(1) – O(1) | 1.4369 |
| Pb (1) - F(3)#7 | 2.7524 | B(3) – O(1) | 1.4315 |
| Pb (1) - F(1)#9 | 2.6283 | B(3) – O(3) | 1.5286 |
| Pb (1) - F(3)#10 | 2.9135 | B(3)#11 – O(3) | 1.5543 |
| Pb (1) - F(1)#11 | 2.7170 | B(1)#12 – O(3) | 1.5225 |
| Pb (1) - F(1)#12 | 2.3825 | B(1)#8 – O(5) | 1.4376 |
| Pb (1) - O(1) | 2.9725 | B(3)#11 – O(5) | 1.4483 |
| Pb (1) - O(5)#7 | 2.6930 |  |  |
| Pb (1) - O(1)#8 | 2.9609 |  |  |

| Bond | Angels (deg) | Bond | Angels (deg) |
| --- | --- | --- | --- |
| F(1)#6 - Pb(1) -O(1) | 71.39 | F(1)#11 -Pb(1) -O(5)#7 | 159.82 |
| F(3)#7 -Pb(1) -O(1) | 57.88 | F(1)#12 -Pb(1) -O(5)#7 | 82.85 |
| O(1) -Pb(1) -O(5)#7 | 82.62 | F(1)#9 -Pb(1) -O(1)#8 | 72.27 |
| O(1) -Pb(1) -O(1)#8 | 97.11 | F(3)#10 -Pb(1) -O(1)#8 | 56.41 |
| F(1)#9 -Pb(1) -O(1) | 160.9 | F(1)#11 -Pb(1) -O(1)#8 | 96.04 |
| F(3)#10 -Pb(1) -O(1) | 130.07 | F(1)#12 -Pb(1) -O(1)#8 | 123.06 |
| F(1)#11 -Pb(1) -O(1) | 97.09 | F(1)#9 -Pb(1) -F(3)#10 | 57.41 |
| F(1)#12 -Pb(1) -O(1) | 124.37 | F(1)#9 -Pb(1) -F(1)#11 | 69.09 |
| F(1)#6 -Pb(1) -F(3)#7 | 58.93 | F(1)#9 -Pb(1) -F(1)#12 | 74.29 |
| F(1)#6 -Pb(1) -O(5)#7 | 127.23 | F(1)#11 -Pb(1) -F(3)#10 | 124.59 |
| F(1)#6 -Pb(1) -O(1)#8 | 160.61 | F(1)#12 -Pb(1) -F(3)#10 | 66.83 |
| F(1)#6 -Pb(1) -F(1)#9 | 113.81 | F(1)#11 -Pb(1) -F(1)#12 | 113.04 |
| F(1)#6 -Pb(1) -F(3)#10 | 142.82 | F(3) -B(1) -O(1) | 106.38 |
| F(1)#6 -Pb(1) -F(1)#11 | 70.78 | F(3) -B(1) -O(5)#3 | 105.92 |
| F(1)#6 -Pb(1) -F(1)#12 | 76 | F(3) -B(1) -O(3)#11 | 107.72 |
| F(3)#7 -Pb(1) -O(5)#7 | 68.31 | O(1) -B(1) -O(5)#3 | 114.55 |
| F(3)#7 -Pb(1) -O(1)#8 | 128.6 | O(1) -B(1) -O(3)#11 | 109.49 |
| F(1)#9 -Pb(1) -F(3)#7 | 141.07 | O(3)#11 -B(1) -O(5)#3 | 112.33 |
| F(3)#7 -Pb(1) -F(3)#10 | 103.4 | O(1) -B(3) -O(3) | 106.2 |
| F(1)#11 -Pb(1) -F(3)#7 | 128.39 | O(1) -B(3) -O(3)#12 | 107.07 |
| F(1)#12 -Pb(1) -F(3)#7 | 66.83 | O(1) -B(3) -O(5)#12 | 117.7 |
| O(1)#8 -Pb(1) -O(5)#7 | 64.12 | O(3) -B(3) -O(3)#12 | 108.91 |
| F(1)#9 -Pb(1) -O(5)#7 | 105.69 | O(3) -B(3) -O(5)#12 | 105.59 |
| F(3)#10 -Pb(1) -O(5)#7 | 48.57 | O(3)#12 -B(3) -O(5)#12 | 111.03 |

Symmetry transformations used to generate equivalent atoms:

| #1 -1+x,-1+y,z | #2 -1+x,y,-1+z | #3 1+x,y,z |
| --- | --- | --- |
| #4 x,-1+y,z | #5 x,y,-1+z | #6 x,y,1+z |
| #7 x,1+y,z | #8 1+x,y,z | #9 1+x,y,1+z |
| #10 1+x,1+y,z | #11 1-x,-1/2+y,1-z | #12 1-x,1/2+y,1-z |

**Table S13.** Real-space atom-cutting results of SHG coefficients

of MB_2_O_3_F_2_ (M = Pb, Pb_0.75_Ba_0.25_, Pb_0.25_Ba_0.75_, Ba).

| Compound |  | SHG Coefficient (pm/V) | SHG Reducing^a^ |
| --- | --- | --- | --- |
| PbB_2_O_3_F_2_ | original | -2.800 (*d*_22_)  -4.952 (*d*_33_) |  |
|  | cut Pb | -0.780 (*d*_22_)  -2.776 (*d*_33_) | 72%  44% |
|  |  |  |  |
| Pb_0.75_Ba_0.25_B_2_O_3_F_2_ | original | -1.734 (*d*_22_)  -3.022 (*d*_33_) |  |
|  | cut Ba(1) | -1.658 (*d*_22_)  -2.738 (*d*_33_) | 4%  9% |
|  | cut Pb(1) | -1.355 (*d*_22_)  -2.660 (*d*_33_) | 22%  12% |
|  | cut Pb(2) | -1.362 (*d*_22_)  -2.659 (*d*_33_) | 21%  12% |
|  | cut Pb(3) | -1.361 (*d*_22_)  -2.664 (*d*_33_) | 22%  12% |
|  |  |  |  |
| Pb_0.25_Ba_0.75_B_2_O_3_F_2_ | original | -1.117 (*d*_22_)  -2.168 (*d*_33_) |  |
|  | cut Ba(1) | -1.040 (*d*_22_)  -1.881 (*d*_33_) | 7%  13% |
|  | cut Ba(2) | -1.045 (*d*_22_)  -1.881 (*d*_33_) | 6%  13% |
|  | cut Ba(3) | -1.042 (*d*_22_)  -1.888 (*d*_33_) | 7%  13% |
|  | cut Pb(1) | -0.753 (*d*_22_)  -1.891 (*d*_33_) | 33%  13% |
|  |  |  |  |
| BaB_2_O_3_F_2_ | original | -0.809 (*d*_22_)  -1.881 (*d*_33_) |  |
|  | cut Ba | -0.530 (*d*_22_)  -0.732 (*d*_33_) | 34%  61% |

^a^SHG reducing is calculated by formula: SHG(reducing) = 1-SHG(cut)/SHG(original)

**Table S14.** Real-space atom-cutting results of SHG coefficients

of MB_2_O_3_F_2_ (M = Sn, Sn_0.75_Ba_0.25_, Sn_0.25_Ba_0.75_, Ba)

| Compound |  | SHG Coefficient (pm/V) | SHG Reducing^a^ |
| --- | --- | --- | --- |
| SnB_2_O_3_F_2_ | original | 0.938 (*d*_22_)  -0.849 (*d*_33_) |  |
|  | cut Sn | 0.254 (*d*_22_)  -1.956 (*d*_33_) | 73%  -130% |
|  |  |  |  |
| Sn_0.75_Ba_0.25_B_2_O_3_F_2_ | original | -0.786 (*d*_22_)  -0.887 (*d*_33_) |  |
|  | cut Ba(1) | -0.593 (*d*_22_)  -1.091 (*d*_33_) | 25%  -23% |
|  | cut Sn(1) | -0.713 (*d*_22_)  -0.941 (*d*_33_) | 9%  -6% |
|  | cut Sn(2) | -0.722 (*d*_22_)  -0.941 (*d*_33_) | 8%  -6% |
|  | cut Sn(3) | -0.730 (*d*_22_)  -0.672 (*d*_33_) | 7%  24% |
|  |  |  |  |
| Sn_0.25_Ba_0.75_B_2_O_3_F_2_ | original | -0.852 (*d*_22_)  -1.522 (*d*_33_) |  |
|  | cut Ba(1) | -0.779 (*d*_22_)  -1.254 (*d*_33_) | 9%  18% |
|  | cut Ba(2) | -0.778 (*d*_22_)  -1.258 (*d*_33_) | 9%  17% |
|  | cut Ba(3) | -0.777 (*d*_22_)  -1.259 (*d*_33_) | 9%  17% |
|  | cut Sn(1) | -0.395 (*d*_22_)  -0.819 (*d*_33_) | 54%  46% |
|  |  |  |  |
| BaB_2_O_3_F_2_ | original | -0.809 (*d*_22_)  -1.881 (*d*_33_) |  |
|  | cut Ba | -0.530 (*d*_22_)  -0.732 (*d*_33_) | 34%  61% |

^a^SHG reducing is calculated by formula: SHG(reducing) = 1-SHG(cut)/SHG(original)

**REFERENCES**

1. Huang, Y.-Z.; Wu, L.-M.; Wu, X.-T.; Li, L.-H.; Chen, L.; Zhang, Y.-F., Pb_2_B_5_O_9_I: An Iodide Borate with Strong Second Harmonic Generation. *J. Am. Chem. Soc.* **2010**, *132*, 12788.

2. Zhang, W.-L.; Cheng, W.-D.; Zhang, H.; Geng, L.; Lin, C.-S.; He, Z.-Z., A Strong Second-Harmonic Generation Material Cd_4_BiO(BO_3_)_3_ Originating from 3-Chromophore Asymmetric Structures. *J. Am. Chem. Soc.* **2010**, *132*, 1508.

3. Xia, M. J.; Jiang, X. X.; Lin, Z. S.; Li, R. K., “All-Three-in-One”: A New Bismuth–Tellurium–Borate Bi_3_TeBO_9_ Exhibiting Strong Second Harmonic Generation Response. *J. Am. Chem. Soc.* **2016**, *138*, 14190.

4. Luo, M.; Song, Y. X.; Liang, F.; Ye, N.; Lin, Z. S., Pb_2_BO_3_Br: A Novel Nonlinear Optical Lead Borate Bromine with a KBBF-Type Structure Exhibiting Strong Nonlinear Optical Response. *Inorg. Chem. Front.* **2018**, *5*, 916.

5. Song, J.-L.; Hu, C.-L.; Xu, X.; Kong, F.; Mao, J.-G., A Facile Synthetic Route to a New SHG Material with Two Types of Parallel π-Conjugated Planar Triangular Units. *Angew. Chem., Int. Ed.* **2015**, *54*, 3679.

6. Yu, H. W.; Koocher, N. Z.; Rondinelli, J. M.; Halasyamani, P. S., Pb_2_BO_3_I: A Borate Iodide with the Largest Second-Harmonic Generation (SHG) Response in the KBe_2_BO_3_F_2_ (KBBF) Family of Nonlinear Optical (NLO) Materials. *Angew. Chem., Int. Ed.* **2018**, *57*, 6100.

7. Dong, X. Y.; Jing, Q.; Shi, Y. J.; Yang, Z. H.; Pan, S. L.; Poeppelmeier, K. R.; Young, J.; Rondinelli, J. M., Pb_2_Ba_3_(BO_3_)_3_Cl: A Material with Large SHG Enhancement Activated by Pb-Chelated BO_3_ Groups. *J. Am. Chem. Soc.* **2015**, *137*, 9417.

8. Zou, G. H.; Lin, C. S.; Jo, H.; Nam, G.; You, T.-S.; Ok, K. M., Pb_2_BO_3_Cl: A Tailor-Made Polar Lead Borate Chloride with Very Strong Second Harmonic Generation. *Angew. Chem., Int. Ed.* **2016**, *55*, 12078.

9. Zou, G. H.; Huang, L.; Ye, N.; Lin, C. S.; Cheng, W. D.; Huang, H., CsPbCO_3_F: A Strong Second-Harmonic Generation Material Derived from Enhancement via p−π Interaction. *J. Am. Chem. Soc.* **2013**, *135*, 18560.

10. Hellwig, H.; Liebertz, J.; Bohatý, L., Exceptional Large Nonlinear Optical Coefficients in the Monoclinic Bismuth Borate BiB_3_O_6_ (BIBO). *Solid State Commun.* **1998**, *109*, 249.

11. Yu, H. W.; Pan, S. L.; Wu, H. P.; Zhao, W. W.; Zhang, F. F.; Li, H. Y.; Yang, Z. H., A New Congruent-Melting Oxyborate, Pb_4_O(BO_3_)_2_ with Optimally Aligned BO_3_ Triangles Adopting Layered-Type Arrangement. *J. Mater. Chem.* **2012**, *22*, 2105.

12. Cong, R. H.; Wang, Y.; Kang, L.; Zhou, Z. Y.; Lin, Z. S.; Yang, T., An Outstanding Second-Harmonic Generation Material BiB_2_O_4_F: Exploiting the Electron-Withdrawing Ability of Fluorine. *Inorg. Chem. Front.* **2015**, *2*, 170.

13. Luo, M.; Liang, F.; Song, Y. X.; Zhao, D.; Ye, N.; Lin, Z. S., Rational Design of the First Lead/Tin Fluorooxoborates MB_2_O_3_F_2_ (M = Pb, Sn), Containing Flexible Two-Dimensional [B_6_O_12_F_6_]_∞_ Single Layers with Widely Divergent Second Harmonic Generation Effects. *J. Am. Chem. Soc.* **2018**, *140*, 6814.

14. Oseledchik, Y. S.; Prosvirnin, A. L.; Pisarevskiy, A. I.; Starshenko, V. V.; Osadchuk, V. V.; Belokrys, S. P.; Svitanko, N. V.; Korol, A. S.; Krikunov, S. A.; Selevich, A. F., New Nonlinear Optical Crystals: Strontium and Lead Tetraborates. *Opt. Mater.* **1995**, *4*, 669.

15. Li, H. Y.; Wu, H. P.; Su, X.; Yu, H. W.; Pan, S. L.; Yang, Z. H.; Lu, Y.; Han, J.; Poeppelmeier, K. R., Pb_3_B_6_O_11_F_2_: The First Non-Centrosymmetric Lead Borate Fluoride with a Large Second Harmonic Generation Response. *J. Mater. Chem. C* **2014**, *2*, 1704.

16. Lin, Y.; Hu, C.-L.; Mao, J.-G., K_2_Pb_3_(CO_3_)_3_F_2_ and KCdCO_3_F: Novel Fluoride Carbonates with Layered and 3D Framework Structures. *Inorg. Chem.* **2015**, *54*, 10407.

17. Yu, H. W.; Wu, H. P.; Pan, S. L.; Yang, Z. H.; Hou, X. L.; Su, X.; Jing, Q.; Poeppelmeier, K. R.; Rondinelli, J. M., Cs_3_Zn_6_B_9_O_21_: A Chemically Benign Member of the KBBF Family Exhibiting the Largest Second Harmonic Generation Response. *J. Am. Chem. Soc.* **2014**, *136*, 1264.

18. Mutailipu, M.; Xie, Z. Q.; Su, X.; Zhang, M.; Wang, Y.; Yang, Z. H.; Janjua, M. R. S. A.; Pan, S. L., Chemical Cosubstitution-Oriented Design of Rare-Earth Borates as Potential Ultraviolet Nonlinear Optical Materials. *J. Am. Chem. Soc.* **2017**, *139*, 18397.

19. Wu, H. P.; Yu, H. W.; Zhang, W. G.; Cantwell, J.; Poeppelmeier, K. R.; Pan, S. L.; Halasyamani, P. S., Top-Seeded Solution Crystal Growth and Linear and Nonlinear Optical Properties of Ba_4_B_11_O_20_F. *Cryst. Growth Des.* **2017**, *17*, 1404.

20. Yu, H. W.; Wu, H. P.; Pan, S. L.; Yang, Z. H.; Su, X.; Zhang, F. F., A Novel Deep UV Nonlinear Optical Crystal Ba_3_B_6_O_11_F_2_, with a New Fundamental Building Block, B_6_O_14_ Group. *J. Mater. Chem.* **2012**, *22*, 9665.

21. Chen, C. T.; Wu, B. C.; Jiang, A. D.; You, G. M., A New-Type Ultraviolet SHG Crystal——β-BaB_2_O_4_. *Sci. Sin. B* **1985**, *28*, 235.

22. Zhao, S. G.; Gong, P. F.; Bai, L.; Xu, X.; Zhang, S. Q.; Sun, Z. H.; Lin, Z. S.; Hong, M. C.; Chen, C. T.; Luo, J. H., Beryllium-Free Li_4_Sr(BO_3_)_2_ for Deep-Ultraviolet Nonlinear Optical Applications. *Nat. Commun.* **2014**, *5*, 4019.

23. Zhao, S. G.; Yang, X. Y.; Yang, Y.; Kuang, X. J.; Lu, F. Q.; Shan, P.; Sun, Z. H.; Lin, Z. S.; Hong, M. C.; Luo, J. H., Non-Centrosymmetric RbNaMgP_2_O_7_ with Unprecedented Thermo-Induced Enhancement of Second Harmonic Generation. *J. Am. Chem. Soc.* **2018**, *140*, 1592.

24. Mutailipu, M.; Zhang, M.; Wu, H. P.; Yang, Z. H.; Shen, Y. H.; Sun, J. L.; Pan, S. L., Ba_3_Mg_3_(BO_3_)_3_F_3_ Polymorphs with Reversible Phase Transition and High Performances as Ultraviolet Nonlinear Optical Materials. *Nat. Commun.* **2018**, *9*, 3089.

25. Wu, H. P.; Pan, S. L.; Poeppelmeier, K. R.; Li, H. Y.; Jia, D. Z.; Chen, Z. H.; Fan, X. Y.; Yang, Y.; Rondinelli, J. M.; Luo, H. S., K_3_B_6_O_10_Cl: A New Structure Analogous to Perovskite with a Large Second Harmonic Generation Response and Deep UV Absorption Edge. *J. Am. Chem. Soc.* **2011**, *133*, 7786.

26. Mori, Y.; Kuroda, I.; Nakajima, S.; Sasaki, T.; Nakai, S., New Nonlinear Optical Crystal: Cesium Lithium Borate. *Appl. Phys. Lett.* **1995**, *67*, 1818.

27. Ye, N.; Zeng, W. R.; Jiang, J.; Wu, B. C.; Chen, C. T., New Nonlinear Optical Crystal K_2_Al_2_B_2_O_7_. *J. Opt. Soc. Am. B* **2000**, *17*, 764.

28. Wu, Y. C.; Fu, P. Z.; Wang, J. X.; Zu Yan Xu; Zhang, L.; Kong, Y. F.; Chen, C. T., Characterization of CsB_3_O_5_ crystal for ultraviolet generation. *Opt. Lett.* **1997**, *22*, 1840.

29. Zhao, S. G.; Gong, P. F.; Luo, S. Y.; Bai, L.; Lin, Z. S.; Ji, C. M.; Chen, T. L.; Hong, M. C.; Luo, J. H., Deep-Ultraviolet Transparent Phosphates RbBa_2_(PO_3_)_5_ and Rb_2_Ba_3_(P_2_O_7_)_2_ Show Nonlinear Optical Activity from Condensation of [PO_4_]^3–^ Units. *J. Am. Chem. Soc.* **2014**, *136*, 8560.

30. Lin, S. J.; Sun, Z. Y.; Wu, B. C.; Chen, C. T., The Nonlinear Optical Characteristics of a LiB_3_O_5_ Crystal. *J. Appl. Phys.* **1990**, *67*, 634.

31. Wang, Y.; Zhang, B. B.; Yang, Z. H.; Pan, S. L., Cation-Tuned Synthesis of Fluorooxoborates: Towards Optimal Deep-Ultraviolet Nonlinear Optical Materials. *Angew. Chem., Int. Ed.* **2018**, *57*, 2150.

32. Shi, G. Q.; Wang, Y.; Zhang, F. F.; Zhang, B. B.; Yang, Z. H.; Hou, X. L.; Pan, S. L.; Poeppelmeier, K. R., Finding the Next Deep-Ultraviolet Nonlinear Optical Material: NH_4_B_4_O_6_F. *J. Am. Chem. Soc.* **2017**, *139*, 10645.

33. Wang, X. F.; Wang, Y.; Zhang, B. B.; Zhang, F. F.; Yang, Z. H.; Pan, S. L., CsB_4_O_6_F: A Congruent-Melting Deep-Ultraviolet Nonlinear Optical Material by Combining Superior Functional Units. *Angew. Chem., Int. Ed.* **2017**, *56*, 14119.

34. Peng, G.; Ye, N.; Lin, Z. S.; Kang, L.; Pan, S. L.; Zhang, M.; Lin, C. S.; Long, X. F.; Luo, M.; Chen, Y.; Tang, Y.-H.; Xu, F.; Yan, T., NH_4_Be_2_BO_3_F_2_ and γ-Be_2_BO_3_F: Overcoming the Layering Habit in KBe_2_BO_3_F_2_ for the Next-Generation Deep-Ultraviolet Nonlinear Optical Materials. *Angew. Chem., Int. Ed.* **2018**, *57*, 8968.

35. Chen, C. T.; Wang, G. L.; Wang, X. Y.; Xu, Z. Y., Deep-UV Nonlinear Optical Crystal KBe_2_BO_3_F_2_—Discovery, Growth, Optical Properties and Applications. *Appl. Phys. B* **2009**, *97*, 9.

36. Huang, C. M.; Zhang, F. F.; Li, H.; Yang, Z. H.; Yu, H. H.; Pan, S. L., BaB_2_O_3_F_2_: A Barium Fluorooxoborate with a Unique [B_2_O_3_F]^−^ Layer and Short Cutoff Edge. *Chem. Eur. J.* **2019**, *25*, 6693.

37. Han, S. J.; Mutailipu, M.; Tudi, A.; Yang, Z. H.; Pan, S. L., PbB_5_O_7_F_3_: A High-Performing Short-Wavelength Nonlinear Optical Material. *Chem. Mater.* **2020**, *32*, 2172.
